# Epigenetic reprogramming-induced guanidinoacetic acid synthesis promotes pancreatic cancer metastasis and transcription-activating histone modifications

**DOI:** 10.1186/s13046-023-02698-x

**Published:** 2023-06-28

**Authors:** Jinshou Yang, Bo Ren, Jie Ren, Gang Yang, Yuan Fang, Xing Wang, Feihan Zhou, Lei You, Yupei Zhao

**Affiliations:** 1 Department of General Surgery, Peking Union Medical College Hospital, Peking Union Medical College, Chinese Academy of Medical Sciences, Beijing, 100023 People’s Republic of China; 2 Key Laboratory of Research in Pancreatic Tumor, Chinese Academy of Medical Sciences, Beijing, 100023 People’s Republic of China; 3 National Science and Technology Key Infrastructure On Translational Medicine in Peking Union Medical College Hospital, Beijing, 100023 People’s Republic of China

**Keywords:** Metabolomics, Epigenetics, H3K4me3, c-Myc, Metastasis

## Abstract

**Background:**

Pancreatic ductal adenocarcinoma (PDAC) tends to undergo distant metastasis, especially liver metastasis, leading to a poor prognosis. Metabolic remodelling and epigenetic reprogramming are two important hallmarks of malignant tumours and participate in regulating PDAC tumorigenesis and metastasis. However, the interaction between these two processes during PDAC metastasis has not been fully elucidated.

**Methods:**

We performed metabolomics analysis to identify the critical metabolites associated with PDAC liver metastasis and focused on guanidinoacetic acid (GAA). Intracellular GAA content was significantly increased in liver metastatic PDAC cells compared to primary cancer cells in mouse xenograft tumour models. The effects of GAA supplementation and glycine amidinotransferase (GATM) knockdown on PDAC metastasis were assessed by analysing cell migration, filopodia formation, epithelial-mesenchymal transition (EMT), and in vivo metastasis in different cell and animal models. Next, ChIP‒qPCR, 3C‒qPCR, and CRISPRi/dCas9-KRAB experiments were used to validate the “epigenome-metabolome" mechanism. Finally, the results of in vitro approaches, including RNA-seq, CUT&RUN, RT‒qPCR, and western blot analyses, as well as luciferase reporter gene assay and transwell assay, revealed the GAA-c-Myc-HMGA axis and transcription-activating histone modifications reprogramming.

**Results:**

A high level of intracellular GAA was associated with PDAC liver metastasis. GAA could promote the migration, EMT, and liver metastasis of pancreatic cancer cells in vitro and in vivo. Next, we explored the role of GATM-mediated de novo GAA synthesis in pancreatic cancer metastasis. High expression of GATM was positively correlated with advanced N stage in PDAC. Knockdown of GATM significantly reduced the intracellular level of GAA, suppressed EMT, and inhibited PDAC liver metastasis, and these effects were attenuated by GAA supplementation. Mechanistically, we identified the active enhancers looped to the *Gatm* gene locus that promoted GATM expression and PDAC liver metastasis. Furthermore, we found that GAA promoted cell migration and EMT by regulating c-Myc-mediated high mobility group AT-hook protein expression. Moreover, GAA increased the H3K4me3 modification level by upregulating histone methyltransferases, which induced the transcription of metastasis-related genes, including *Myc*.

**Conclusions:**

These findings revealed the critical role of the epigenome-metabolome interaction in regulating PDAC liver metastasis and suggested potential therapeutic strategies targeting GAA metabolism and epigenetic regulatory mechanisms.

**Supplementary Information:**

The online version contains supplementary material available at 10.1186/s13046-023-02698-x.

## Background

Pancreatic ductal adenocarcinoma (PDAC), a highly malignant tumour of the digestive system, accounts for over 85% of all malignant pancreatic exocrine tumours [1]. Only approximately 10% of diagnosed PDAC patients are eligible for radical resection, while most patients are diagnosed with late-stage disease [2]. Notably, PDAC tends to metastasize to the liver, and liver metastasis is found in 35% of newly diagnosed PDAC cases [3]. Among patients with recurrence or metastasis, 47.6% develop liver metastases within six months after surgery [4]. Metastasis results in a poor prognosis in PDAC patients and leads to undesirable therapeutic effects.

Metabolic remodelling and non-mutation-related epigenetic reprogramming are considered important hallmarks of cancer [5]. Recently, the role of crosstalk between these two hallmarks in PDAC progression has attracted researchers’ attention. Oliver et al. reported that PDAC distant metastases coevolved a dependence on the oxidative branch of the pentose phosphate pathway (oxPPP) or activated phosphogluconate dehydrogenase (PGD), which selectively reversed reprogrammed chromatin and promoted N-cadherin expression [6, 7]. In our previous work, we developed high-resolution 3D maps of the epigenomic landscape during pancreatic cancer metastasis. We found that enhancer-promoter loop reprogramming could upregulate lipase C (LIPC), a key enzyme involved in lipoprotein metabolism, to promote PDAC metastasis [8]. Moreover, *Kras* mutation in PDAC facilitated the expression of ATP-citrate lyase (ACLY) to induce acetyl-CoA accumulation, which promoted acinar-ductal metaplasia during pancreatic carcinogenesis via the mevalonate pathway and histone acetylation [9]. These studies highlight the importance of the interaction between the epigenome and metabolome during PDAC progression.

To further explore the crosstalk between epigenetic reprogramming and metabolic remodelling during PDAC metastasis, we performed metabolomics analysis and integrated the acquired data with our previous epigenetic data [8]. We found that guanidinoacetic acid (GAA) was closely related to liver metastasis in PDAC. Glycine amidinotransferase (GATM), also called L-arginine:glycine amidinotransferase (AGAT), is the rate-limiting enzyme of de novo GAA synthesis. We identified a candidate enhancer region that looped to the *Gatm* gene promoter, providing potential evidence of an “epigenome-metabolome” interaction program active during PDAC metastasis. GATM has been reported to be associated with high aggressiveness in sarcoma and renal carcinoma [10, 11]. However, the roles and mechanisms of GATM and GAA in pancreatic cancer metastasis are incompletely elucidated. Hence, we hypothesized that enhancer-promoter looping could mediate GATM overexpression and enhance de novo GAA biosynthesis, which would in turn promote PDAC liver metastasis.

## Methods

### Cell culture and transfection

Normal human pancreatic epithelial cells (HPNE) and various pancreatic adenocarcinoma cell lines, including PANC-1, MIA PaCa-2, BxPC-3, Capan-1, CFPAC-1, were obtained from the American Type Culture Collection (ATCC). PANC-1-IN, or PANC-1 Invasion-8 was constructed previously by Doc. Gang Yang previously [12]. Pancreatic cancer cells were cultured in DMEM, RPMI 1640, or IMDM medium with high glucose levels and supplemented with 10–20% fetal bovine serum (FBS) and 1% penicillin–streptomycin. All cell lines were maintained at 37℃ with 5% CO_2_, routinely tested for mycoplasma by PCR and authenticated through high-resolution small tandem repeats (STR) profiling. The siRNAs used in this study were purchased from RiboBio Co., LTD (Guangzhou. China) (GATM: 5’-GGACGAACCTTGACAGGAT-3’; MYC: 5’-GAGGAGACATGGTGAACCA-3’; KMT2A: 5’-CUCUCCUCUCAAGUCUAAGUU-3’; KMT2C: 5’-UGGUGUCAAAAAGAGAAAAAG-3’; KMT2D: 5’-CACAUGGAGUGCGAAAUUATT-3’; SETD1A: 5’-GUUUGAACAGAUGACCAUCCU-3’; SETD1B: 5’-AUGUGAAGGUGGAAUCAGAGA-3’; SLC6A6: 5’- GTTTCTCATACCGTATTTT-3’; SLC6A8: 5’-AGCCGCTGGTCTACAACAA-3’). Plasmid GV366 (CMV-MCS-HA-SV40-Neomycin) was used to clone wild-type *Hmga1*, *H*mga2 and *Myc* genes. The control plasmid was named CON237. Transient transfection was performed using Lipofectamine™ 3000, following the corresponding protocol.

### Lentiviral vector and infection

The shRNA (5’-GGACGAACCTTGACAGGAT-3’) targeting *Gatm* was packaged into lentiviral vector GV344 (hU6-MCS-Ubiquitin-firefly_Luciferase-IRES-puromycin) obtained from GENECHEM (Shanghai, China). Lenti-EF1a-dCas9-KRAB-Puro virus was commercially obtained from GenePharma Technology Co., Ltd. (Shanghai, China). The sgRNA against specific enhancers E4, E5, E6 of the *Gatm* gene (sg10947: ATGTACCTAAGATAGCGGT; sg10948: GCAGACACAGCAGACGCGT; sg10949: GCGGCCACGCCTTACCGAA) were packaged into the lentiviral vector GV391 (U6-sgRNA-SV40-Neomycin) obtained from GENECHEM. Pancreatic cancer cells were infected following the manufacturer’s protocol. After three days, pancreatic cancer cells were selected using medium containing 2 μg/mL puromycin or 800 μg/mL G418 and were maintained in medium containing 1 μg/mL puromycin or 200 μg/mL G418.

### RNA extraction and PCR assays

Total RNA was extracted from samples using the RNA-Quick Purification Kit (ES Science, RN001) and the RNA concentration was measured using the Thermo Scientific™ NanoDrop™ spectrophotometer. cDNA was synthesized using the TAKARA PrimerScript™ reagent Kit with gDNA eraser (RR047A). RT-qPCR was performed on the QuantStudio3 real-time PCR instrument using PowerUp™ SYBR™ Green Master Mix (AppliedBiosystem, 01,076,912). All used primers are listed in Supplementary materials_0^7^. The expression of each gene was determined using the threshold cycle (Ct) values, and the relative expression level was calculated through the 2^−ΔΔCt^ method, normalized to the endogenous reference GAPDH. Three replicate wells were analyzed per group.

### Western blotting

Whole cell lysate was prepared in RIPA lysis buffer (APPLYGEN, C1053) supplemented with protease inhibitors (AbMole, M5293) and phosphatase inhibitor (AbMole, M7528) in a 1.5 mL EP tube on ice. After centrifugation at 12,000 rpm for 10 min, the supernatants were collected and protein concentrations were measured using the Pierce™ BCA Protein Assay Kit (Thermo Fisher Scientific, UE284362). Protein samples were mixed with 5 × loading buffer (GeneStar, 20BB01), separated by 10% SDS-PAGE, and transferred to a NC membrane (0.22 μm or 0.45 μm pore-size). The membranes were blocked with 5% non-fat milk in TBST buffer for one hour, and incubated with primary antibodies overnight at 4 ℃. Finally, the membranes were incubated with HRP-conjugated anti-rabbit or anti-mouse antibodies for one hour at room temperature, and the target protein bands were visualized using the SuperSignal™ West Dura Extended Duration Substrate (Thermo Fisher Scientific, VH311118). All the used antibodies and working dilution are detailed in Supplementary Materials_0^7^.

### Transwell assay

3.0 × 10^4^ PANC-1 cells, 2.0 × 10^4^ PANC-1-IN cells, 6.0–8.0 × 10^4^ BxPC-3/CFPAC-1 cells, and 2.0 × 10^5^ Capan-1 cells were suspended with FBS-free corresponding medium and plated in the upper chamber (Costar, 3422). For the Capan-1 assay, the upper chambers were pre-coated with 1:20 diluted Matrigel. The lower chamber was filled with complete medium containing 10% FBS. After 24 h, the migrated cells on the lower side surface of the filter were fixed by methanol for 20 min and stained with 0.5% crystal violet solution for 20 min. The membranes were then washed and dried, we calculated the number of stained cells of five high-power fields in microscope per chamber filter. Three independent filters were analyzed per group.

### Cell proliferation assay

1500–3000 cells of PANC-1, MIA PaCa-2, PANC-1-IN, BxPC-3, CFPAC-1, and 5000 cells of Capan-1 were plated in the 96-wells plates containing appropriate medium with 10% FBS. The Cell Counting Kit-8 (CCK8, DOJINDO, CK04) was used after three days of treatment with different mediums to analyse OD630 and OD450 values (Fig. S1). For investigation of PANC-1-IN, Capan-1, BxpC-3 and CFPAC-1 cell proliferation after GATM knockdown and GAA supplementation, the Sulforhodamine B (SRB) assay was used (Fig. S3). After fixation by 10% trichloroacetic acid and staining with 4% SRB solution, absorbance was measured at OD564 using 10 nM tris-base. Six replicate wells were analysed per group.

### Luciferase reporter assay

To examine the promoter activity of *Hmga1* and *Hmga2*, PANC-1-IN cells (1 × 10^5^/well) were plated in 12-well plates and transfected with expression plasmids CMV-MYC-HA-SV40-Neomycin or the vector GV366 (CMV-HA-SV40-Neomycin) (1 μg/well) using Lipofectamine 3000. Additionally, PANC-1-IN cells were treated with 1 mM GAA solution or PBS buffer solution. The following day, GV238-*Hmga1* or *Hmga2* promoter-firefly luciferase reporter plasmid was co-transfected with renilla luciferase plasmid. After 48 h, the firefly and renilla luciferase activities were measured using the Dual Luciferase Reporter Gene Assay Kit (YEASEN, Cat NO. 11402ES) and a multifunctional microplate reader (Tristar LB 942, Berthold, Germany) according to the manufacturer’s intructions.

### Untargeted metabolomics

For each sample, 1 × 10^7^ cells were collected from a 20 cm plate and quickly frozen in liquid nitrogen. Six independent samples were prepared for each group. 1000 μL extract solution (acetonitrile: methanol: water = 2: 2: 1) containing an internal standard was added, and the samples were homogenized and sonicated. After incubation at -40 ℃ for 1 h, the sample were centrifuged with 12,000 rpm for 15 min. Then, 800 μL of the supernatant was transferred to a fresh tube and dried in a vacuum concentrator. The dried samples were reconstituted in 100 μL acetonitrile solution and subjected to 10 min sonication. After centrifugation at 13,000 rpm for 15 min, 75 ul of the supernatant was transferred to a fresh glass vial for LC–MS analysis. The LC–MS/MS analysis of HPNE cells, PANC-1 cells and Capan-1 cells was conducted using a 1290 Infinity series UHPLC System (Agilent Technologies) and TripleTOF 6600 mass spectrometry (AB Sciex) by BIOTREE BIOMEDICAL TECHNOLOGY CO., LTD (Shanghai, China). The global metabolite profiling of PRM cells and LMT cells was accomplished using an LC–ESI–MS/MS system (UPLC, ExionLC AD; MS, QTRAP® System) was accomplished by Metware Biotechnology Inc (Wuhan, China).

### Metabolites quantitative measurement

The process of samples preparation and metabolites extraction for targeted metabolomics was the same as that for untargeted metabolomics. A mixed working standard solution with a final concentration of 10 mmol/L was prepared by dissolving or diluting each standard substance individually, and an aliquot of each was transferred to a 10 mL flask. A series of calibration standard solutions were then prepared by stepwise dilution of this mixed standard solution. LC-MRM-MS analysis (including GAA, creatine, SAM, acetyl-CoA, etc.) was performed using an EXIONLC system (Sciex) and an SCIEX 6500 OTRAP + triple quadrupole mass spectrometer by BIOTREE BIOMEDICAL TECHNOLOGY CO., LTD (Shanghai, China). The y value of the calibration curves represents the peak areas of the analyte and x represents the concentration (nmol/L) for the analyte. The final concentration (nmol/L) of each target analyte was calculated by multiplying the calculated concentration (nmol/L) by the dilution factor. Additionally, the UPLC-MS/MS analysis, which included quantitative measurement of plasma GAA and creatine, relative quantitative measurement of the plasma phospho-creatine (Fig. 2), and quantitative measurement of GAA in PANC-1 PRM cells and LMT cells were conducted using Waters IClass UPLC-AB Sciex Triple Quad 5500 + and Thermo Scientific Ultimate 3000/TSQ Altis in Dalian Institute of Chemistry Physics, Chinese Academy of Sciences.

### RNA sequencing and analysis

Total RNA was extracted from pancreatic cancer cells using TRIzol reagent (Thermo Fisher Scientific, 15,596–018). NEBNext® Ultra™ RNA Library Prep Kit for Illumina (NEB, E7530L) was used for sequencing library construction, and Illumina NovaSeq platform with PE150 strategy was used for sequencing. The reads were mapped to the human hg19 genome by HiSAT2. Gene expression was quantified using HTSeq and normalized by FPKM method. DESeq2 was used to identify differentially expressed genes with adj. *p* ≤ 0.05 (Benjamini and Hochberg's method) and |log2(fold change) |≥ 1. Significantly differential pathways were identified using the limma R package by calculating the ssGSEA score of Hallmarks gene sets (http://www.gsea-msigdb.org/gsea/msigdb/) in each sample of RNA-seq.

RNA-seq data of liver metastatic cancer cells and primary cancer cells were obtained from our previously published work (GSE149103) [8]. Correlation analysis of top 1000 ~ differentially expressed genes ranked by* P* value with untargeted metabolomics data was performed using “spearman” algorithm. GO enrichment analysis of DEGs and Venn analysis was performed using R package. Gene set variation analysis (GSVA) method was performed to compare the gene sets enrichment score and overall surviva [13, 14]. Patients in TCGA cohort (*n* = 176) were stratified into low enrichment (GSVA score ≤ median) and high enrichment (GSVA score > median) groups.

### Hi-C and ChIP-seq analysis

Hi-C and ChIP-seq data were obtained from GSE149103 [8]. For data processing, the normalized 5-kb resolution Hi-C matrices were created by HiC-Pro [15]. Significant Hi-C contacts were identified by Juicer software [16]. Visualization of Hi-C and ChIP-seq was performed by WashU Epigenome Browser.

### 3C-PCR

3C-qPCR were performed according to the published protocol [17] and described previously [8]. In brief, 1 × 10^7^ cells (PANC-1, MIA PaCa-2, BxPC-3, PANC-1-IN, Capan-1) were collected, crosslinked and lysed respectively. Hind III restriction enzyme was used to digest genomic DNA. Candidate primers constant primer were designed within 50 bp upstream of Hind III restriction site. The 3C ligation products were quantitatively analyzed by SYBR Green-based PCR, with GAPDH internal primers as negative control for normalization. Primer sequences are listed in Supplementary materials_0^7^.

### ChIP-qPCR

A SimpleChIP®Plus Enzymatic Chromatin IP Kit (Cell Signaling Technology, 9005) was used to perform the ChIP experiment. A total of 4 × 10^6^ cells were crosslinked and prepared as an IP sample. After nuclei preparation and chromatin digestion, the lysates were centrifugated at 9400 g for 10 min at 4 ℃. Supernatant were diluted to 500 μL by 1 × ChIP buffer containing protease inhibitor, and 10 μL of antibody targeting H3K27ac was added followed by incubation, elution, reverse crosslinking, and purification steps. The target DNA fragment was then collected. The modification level of H3K4me3 and H3K27ac was analyzed using qPCR, and presented as percent input, which was calculated as 2% × 2^(C[T]2%input sample−C[T]IP sample)^. Primer sequences are listed in Supplementary materials_0^7^.

### CUT&RUN assay

CUT&RUN experiments were conducted using CUT&RUN Assay Kit (Cell Signaling Technology, 86,652). In brief, one hundred thousand fresh cells were collected and immobilized on Concanavalin A magnetic Beads, allowing for subsequent buffer and reagent exchanges. Cell membranes were permeabilized with digitonin to facilitate the entry of primary antibody and pAG-MNase fusion enzyme into the cell nuclei. The addition of Ca^2+^ activated the pAG-MNase, which gently cleaved and liberated the desired chromatin fragments, enabling them to diffuse away from the genomic chromatin, exit the cell, and enter the supernatant. DNA was purified using DNA purification spin columns. The modification level of H3K4me3 and H3K4me1 was analyzed using qPCR and presented as percent input, which was calculated as 2% × 2^(C[T]2%input sample−C[T]IP sample)^. Primer sequences are listed in Supplementary materials_0^7^. The DNA library was constructed using VAHTS Universal DNA Library Prep Kit for Illumina V3 (Vazyme#ND607-01). The genome-wide analysis was then performed on the Illumina sequencing platform with a read length of PE150.

### Methylation specific PCR

Pancreatic cancer cells were digested and collected from 10 cm plates. Whole genomic DNA was extracted using TIANamp Genomic DNA Kit (TIANGEN, DP304-02). 2 μg of DNA was diluted to 50 μL with ddH_2_O in a 1.5 mL tube, followed by the addition of 5.5 μL of 3 M NaOH solution and incubated for 30 min at 42 ℃. 30 μL of 10 M hydroquinone (MACKLIN, C12642868) and 520 μL of 6 M sodium bisulfite (Sigma-Aldrich, 243,973-100G) were added to the liquid mixture, incubated in the dark for 16 h at 50 ℃, and then the modified DNA was purified using the Universal DNA Purification Kit (TIANGEN, DP214-03). The methylation status of *Gatm* promoter was qualitatively analyzed through PCR and DNA gel electrophoresis. Methylation-specific primers and unmethylation-specific primers were designed using MethPrimer 2.0.

### Enhancer targeting CRISPR Epigenetic editing

Short guide RNAs (sgRNAs) used in CRISPR interference/activation (CRISPRi/a) were designed using CRISPOR (http://crispor.tefor.net/) [18]. The gRNAs (Supplementary materials_0^7^) targeting GATM promoter and remote enhancers were cloned into the phU6-gRNA vector (MiaoLingbio, P1715) with *Bbs*I restriction enzymatic site. Briefly, 1 μg of plasmid was digested with *Bbs*I (NEB, R0539S) at 37 ℃ for 30 min, and the digested plasmid was purified using the QIAquick Gel Extraction Kit and elute in EB buffer. 50 ng of *Bbs*I digested plasmid and phosphorylated and annealed sgRNA oligos were ligated using quick ligase (NEB, M2200S). Pancreatic cancer cells stably expressing the dCas9-KRAB fusion protein were transfected with the recombinant vectors using Lipofectamine™ 3000 (Invitrogen, L3000015). After 48 h post-transfection, cells were harvested and GATM expression was assessed using RT-qPCR and western blotting.

### Xenograft models by orthotopic injection and splenic injection

The orthotopic injection xenograft tumor model was established as previously described [8]. Briefly, mice were anesthetized with isoflurane and underwent surgery to open the abdomen. 5 × 10^6^ pancreatic cancer cells expressing luciferase were injected into the pancreas, and the mice abdomen was closed using sterile suture. In splenic injection mouse model, 1 × 10^6^ cancer cells were injected into the spleen. The dietary intervention group was provided with fodder containing 1% guanidinoacetic acid (Sigma-Aldrich, G11608). Tumor growth and invasion were evaluated in vivo using the IVIS imaging system every 2 weeks. After 4–6 weeks of model establishment, mice were anesthetized and injected intraperitoneally with 15 mg/mL D-Luciferase potassium salt (10μL/g). The livers and lungs were dissected within 7–10 min after substrate injection to evaluate the distant metastasis using ex vivo imaging. Meanwhile, blood of mice was collected into 1.5 mL tubes with heparin. The primary tumor, liver and lung tissues were fixed in 4% poly-formaldehyde solution overnight, then dehydrated and paraffin-embedded in paraffin. Paraffin-embedded tissues were sectioned at 4 μm thickness for H&E staining. All animal experiments were conducted according to the institutional ethical guidelines of PUMCH (Beijing, China).

### Clinical specimens and immunohistochemistry

Eighty-one postoperative pancreatic cancer specimens were obtained from the Department of Pathology at PUMCH. All patients did not receive neoadjuvant therapy. A tissue microarray consisting of seventy-two primary pancreatic cancer tissues was obtained from the Department of General Surgery of PUMCH. Detailed clinical and pathological features of these patients were listed in Supplementary materials_0^6^. The study was approved by the ethics committee of PUMCH, and written informed consent was obtained from all donors.

Tissue section were deparaffinized, rehydrated and boiled for 20 min in citrate antigen retrieval solution. Slides were blocked using 10% goat serum for 30 min and incubated with antibody at room temperature for two hours. Sections were then incubated with HRP-conjugated secondary antibody for 30 min, DAB for 20 s to 1 min, followed by hematoxylin staining. Finally, sections were dehydrated and mounted in neutral resins. Two experienced pathologists independently assessed the IHC results. Staining intensity were graded as 0 (negative), 1 (low), 2 (moderate), 3 (high), while staining extent was scored from 0 to 100%. The final IHC score equals to intensity score multiplied by percentage score.

### Data mining in TCGA, GEO and CCLE

A total of 181 pancreatic samples with RNA-seq data and survival data were extracted from the National Cancer Institute GDC Data Portal (https://portal.gdc.cancer.gov/). Gene expression data from GSE42952 [19] was used for GSEA of arginine and proline pathway genes between primary tumor and metastases. The data from GSE71729 [14] was downloaded and used for gene expression analysis among normal pancreas, primary tumors, and liver metastases. The data of GSE71729, GSE78229, GSE62452 was used for Kaplan–Meier survival analysis by using R packages. Gene mutation, copy number alteration and DNA methylation of GATM were evaluated using cBioPortal (http://www.cbioportal.org/) and Xena (https://xenabrowser.net/).

### Statistics analysis

Statistics analysis was performed using IBM SPSS Statistics 21 and GraphPad Prism 8. For clinical data, Pearson’s χ^2^ test was used to analyze the relationship between GATM expression and clinicopathological characteristics. Survival analysis of publicly available data was conducted using Kaplan–Meier method, and comparisons were made using log-rank test. In vitro and in vivo experiments, two-tailed Student’s test, one-way ANOVA or two-way ANOVA was used to calculate statistical significance between groups. In vitro experiments were independently performed three times. Significance values are represented as * *P* < 0.05, ** *P* < 0.01, *** *P* < 0.001, **** *P* < 0.0001.

## Results

### A high level of intracellular GAA is associated with PDAC liver metastasis

**Fig. 1:**
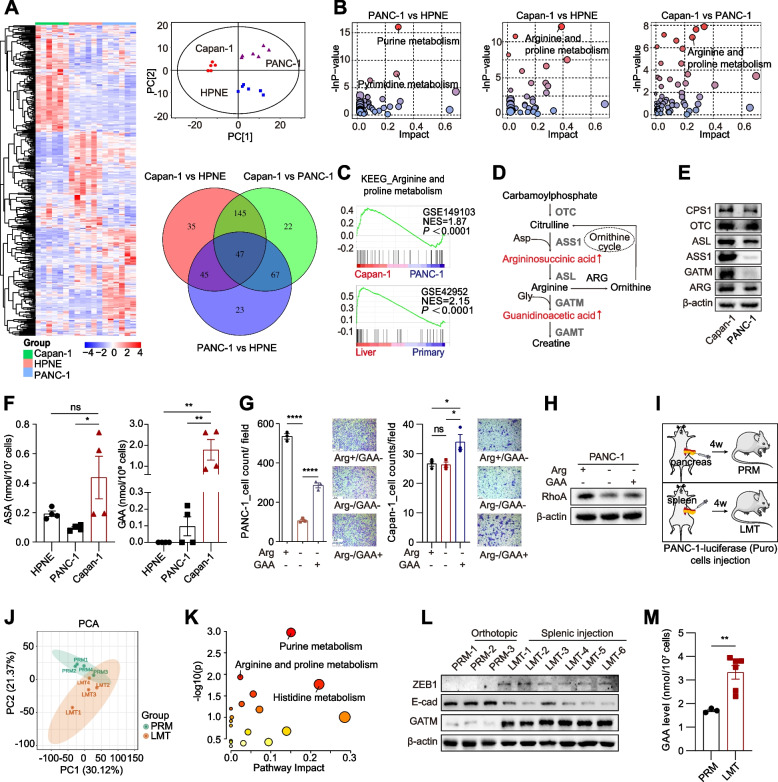
A high level of intracellular GAA was associated with PDAC liver metastasis. **A** Hierarchical clustering analysis of 595 differential metabolites among normal (HPNE), primary cancer cells (PANC-1) and liver metastatic cancer cells (Capan-1). The color shade represents the relative expression level of metabolites. Principal component analysis (PCA) of original data consisting of 3281 Peaks (upper right). Venn analysis of differential metabolites between every two groups of pancreatic cancer cells (lower right). **B** Metabolic pathway analysis of differential metabolites between every two groups. The horizontal axis and the bubble size represented the impact factor of this pathway. The vertical axis and the bubble color represented the *p* value in the enrichment analysis. **C** GSEA showing arginine and proline metabolism enhanced in Capan-1 cells versus PANC-1 (GSE149103), and in liver metastases versus primary tumor (GSE42952). **D** Schematic of ornithine cycle and arginine metabolism. **E** Western blot showing expression of enzymes in arginine metabolism pathway. **F** Quantitative measurement of intracellular metabolites by LC–MS/MS. **G** Transwell migration assay of pancreatic cancer cells cultured in different conditions. GAA (1 mM). 100 × magnifying power. **H** Western blot showing RhoA expression in pancreatic cancer cells. **I** Schematic of mice models of orthotopic injection and splenic injection. **J** PCA analysis of the metabolites in PRMs and LMTs. **K** Metabolic pathway analysis of different metabolites between PRMs and LMTs. **L** Western blot showing GATM expression in PRM cells and LMT cells. **M** Quantitative measurement of intracellular GAA in PANC-1 PRM cells and LMT cells by LC–MS/MS. Data in (**F**-**G**) and (**M**) are presented as mean ± SEM by ordinary one-way ANOVA (Tukey’s multiple comparisons test) and unpaired student t test. * *P* < 0.05, ** *P* < 0.01, *** *P* < 0.001, **** *P* < 0.0001

We previously generated high-resolution 3D maps of the epigenome landscape during pancreatic cancer metastasis by using pancreatic normal ductal epithelial cells (HPNE), primary pancreatic cancer cells (PANC-1) and liver metastatic cancer cells (Capan-1) [8]. PANC-1 cells and Capan-1 cells have different metastatic abilities in vivo [20]. Model mice implanted with Capan-1 cells are more likely to develop distant metastasis than those implanted with PANC-1 cells [21, 22]. To explore the role of the crosstalk between metabolic remodelling and epigenetic reprogramming during PDAC metastasis, we collected cell samples to perform metabolomics analysis approximately concurrently with Hi-C and ChIP-seq. In this effort, an untargeted metabolomics approach was used to analyse the abovementioned three types of cells, and the acquired data were integrated with previously obtained epigenetic data. We identified the differentially abundant metabolites between every set of two groups (Supplementary materials_0^1^). The largest number of differentially abundant metabolites was observed between liver metastatic cells and primary cancer cells (Fig. 1A). Further analysis showed that arginine and proline metabolism was one of the most differentially enriched metabolic pathways between liver metastatic cancer cells and primary cancer cells (Fig. 1B). Gene set enrichment analysis (GSEA) of RNA-seq data also showed higher enrichment scores of the arginine and proline metabolic pathway in liver metastatic cells than in the pancreatic primary cancer cells (Fig. 1C). The levels of argininosuccinic acid (ASA) and GAA, which are involved in the arginine and proline metabolic pathways, were higher in Capan-1 cells than in PANC-1 cells (Fig. 1D). Through integrated metabolome and transcriptome analyses, we identified differentially expressed genes (DEGs) related to ASA and GAA (Supplementary materials_0^3^). These DEGs were enriched in pathways related to the cancer metastasis, such as GTPase regulator activity (Fig. S1A-B).

The rate-limiting enzymes, argininosuccinate synthetase 1 (ASS1) and GATM, which catalyse the de novo synthesis of arginine and GAA, were expressed at higher levels in Capan-1 cells than in PANC-1 cells (Fig. 1E). Next, the contents of ASA and GAA were quantitatively measured and were found to be significantly increased in Capan-1 cells compared to PANC-1 cells (Supplementary materials_0^2^) (Fig. 1F). Arginine depletion was reported to inhibit the migration of pancreatic cancer cells that lacked ASS1 expression and were dependent on exogenous arginine [23–25]. Indeed, arginine depletion significantly inhibited PANC-1 cell migration, which was partially rescued by GAA supplementation (Fig. 1G). However, arginine depletion did not suppress the migration of Capan-1 cells, although GAA supplementation significantly promoted Capan-1 cell migration (Fig. 1G). This difference might be attributed to the higher expression levels of ASS1 and GATM in Capan-1 cells to ensure an abundant supply of intracellular arginine and GAA. We also found that arginine depletion obviously suppressed RhoA expression, which was partially rescued by subsequent GAA supplementation in PANC-1 cells (Fig. 1H). We found consistent results in BxPC-3 and MIA PaCa-2 cells, which have high and low levels, respectively, of these two enzymes (Fig. S1C-F). Thus, arginine might promote pancreatic cancer cell migration via GAA production. Additionally, arginine depletion significantly inhibited the proliferation of pancreatic cancer cells regardless of the ASS1 expression level (Fig. S1E). However, GAA supplementation failed to rescue the proliferation of Capan-1 cells and BxPC-3 cells, indicating the role of arginine in promoting proliferation independent of GAA (Fig. S1F).

To fully validate the association of GAA with PDAC liver metastasis, we established mouse models using orthotopic injection and splenic injection of PANC-1-luciferase cells expressing a puromycin (puro) selection marker (Fig. 1I). After four weeks, we isolated primary pancreatic tumour cells (PRMs) and liver metastatic tumour cells (LMTs). Global metabolite profiling of PRMs and LMTs was then accomplished. These two groups had significant differences in metabolite distribution (Fig. 1J) (Supplementary materials_0^1^). Arginine and proline metabolism was one of the most differentially enriched pathways between PRMs and LMTs (Fig. 1K). Correspondingly, the expression of GATM and ZEB1 was obviously increased in LMTs compared to PRMs (Fig. 1L). In addition, the GAA content was significantly increased in LMTs compared to PRMs (Fig. 1M).

### GAA promotes PDAC liver metastasis

**Fig. 2:**
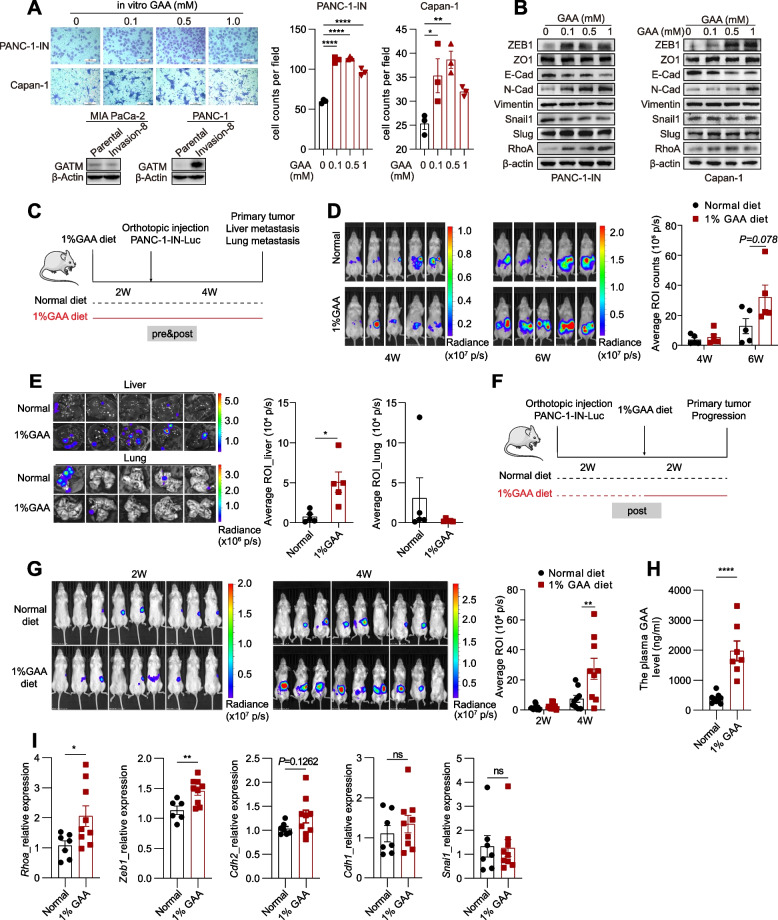
GAA promoted liver metastasis of PDAC. **A** Transwell migration assay of pancreatic cancer cells cultured in medium with 0 mM, 0.1 mM, 0.5 mM and 1 mM GAA (upper). 200 × magnifying power. Western blot showing the expression of GATM in pancreatic cancer cells (lower). **B** Western blot showing expression of EMT markers. **C** PDAC orthotopic mouse model experiments with 6 weeks GAA diet regimen (pre&post). **D** IVIS bioluminescence images and quantification of PDAC xenograft tumor progression. **E** IVIS bioluminescence images and quantification of resected livers and lungs. **F** PDAC orthotopic mouse model experiments with 2 weeks GAA diet regimen (post). **G** IVIS bioluminescence images and quantification of PDAC xenograft tumor progression. **H** Quantitative measurement of the plasma GAA by LC–MS/MS. **I** RT-qPCR showing the expression of EMT marker genes in primary tumors of mice receiving different diets. Data in (A) are presented as mean ± SEM by ordinary one-way ANOVA (Dunnett’s multiple comparisons test). Data in (D-E, G-I) are presented as mean ± SEM by unpaired t test. * *P* < 0.05, ** *P* < 0.01, *** *P* < 0.001, **** *P* < 0.0001

Whether GAA plays a critical role in promoting PDAC metastasis remains unknown. Therefore, we treated several pancreatic cancer cell lines with different concentrations of GAA and found that their migration ability was significantly augmented and EMT was enhanced (Fig. 2A-B; Fig. S2A-C). We previously established a highly metastatic human pancreatic cancer cell line, PANC-1 Invasion-8 [12], which was derived from PANC-1 cells and renamed PANC-1-IN. Compared to the parental cells, PANC-1-IN cells had a higher expression level of GATM (Fig. 2A). After GAA treatment, the expression of ZEB1, N-cadherin, and RhoA was increased, and that of E-cadherin, a key epithelial marker was downregulated (Fig. 2B; Fig. S2C). GAA in the blood circulation can be taken up into GAA-demanding tissues via several transporters, such as the creatine transporter (SLC6A8), taurine transporter (SLC6A6), and γ-aminobutyric acid transporter (SLC6A13), or by passive diffusion [26]. We found that *SLC6A6* and *SLC6A8* had higher expression levels in liver metastases than in normal pancreatic tissues and primary tumours (Fig. S2D). However, knockdown of SLC6A6 and SLC6A8 did not diminish the promoting effect of GAA on pancreatic cancer cell migration (Fig. S2E), possibly because these cells have a greater dependence on passive diffusion of extracellular GAA or de novo GAA synthesis than on the expression of SLC6A6 and SLC6A8.

To evaluate the promoting role of dietary GAA supplementation during pancreatic cancer metastasis in vivo, we established an orthotopic injection mouse model in four-week-old NOD. Cg-Prkdc^scid^ Il2rg^tm1Vst^/Vst (NPG) mice. Mice in the treatment group were fed a diet containing 1% GAA for 2 weeks prior to orthotopic injection of PANC-1-IN cells with stable luciferase expression (pre). The GAA diet regimen was continued for up to 4 additional weeks (post) (Fig. 2C). We found an increasing trend in the bioluminescence signal in mice in the GAA diet group compared to the control group (Fig. 2D). We then harvested the livers and lungs from the mice and conducted ex vivo bioluminescence imaging. The number of liver metastases was significantly increased in the GAA diet group compared to the control group, but the number of lung metastases was nonsignificantly decreased (Fig. 2E). To minimize the impact of the GAA diet on whole-body metabolism in mice, we initiated the GAA diet regimen 2 weeks after the injection of PANC-1-IN cells (Fig. 2F). We found a significant increase in the bioluminescence signal in mice in the GAA diet group compared to mice in the control group in the fourth week (Fig. 2G). Next, we quantified the metabolites in mouse plasma and found an obvious increase in the GAA content in the treatment group (Fig. 2H). The expression of the EMT markers *Rhoa* and *Zeb1* in primary tumours was significantly higher in mice in the GAA diet group than in mice in the control group (Fig. 2I).

In addition, GAA diet feeding did not result in a difference in primary tumour growth but led to a reduction in liver weight (Fig. S2G-H, J-K). The obvious decrease in body weight, which occurred in the presence of both GAA intake and tumour growth in the mice, seemed to be a result of cachexia and not GAA supplementation (Fig. S2F, I).

### Knockdown of GATM suppresses PDAC liver metastasis

**Fig. 3:**
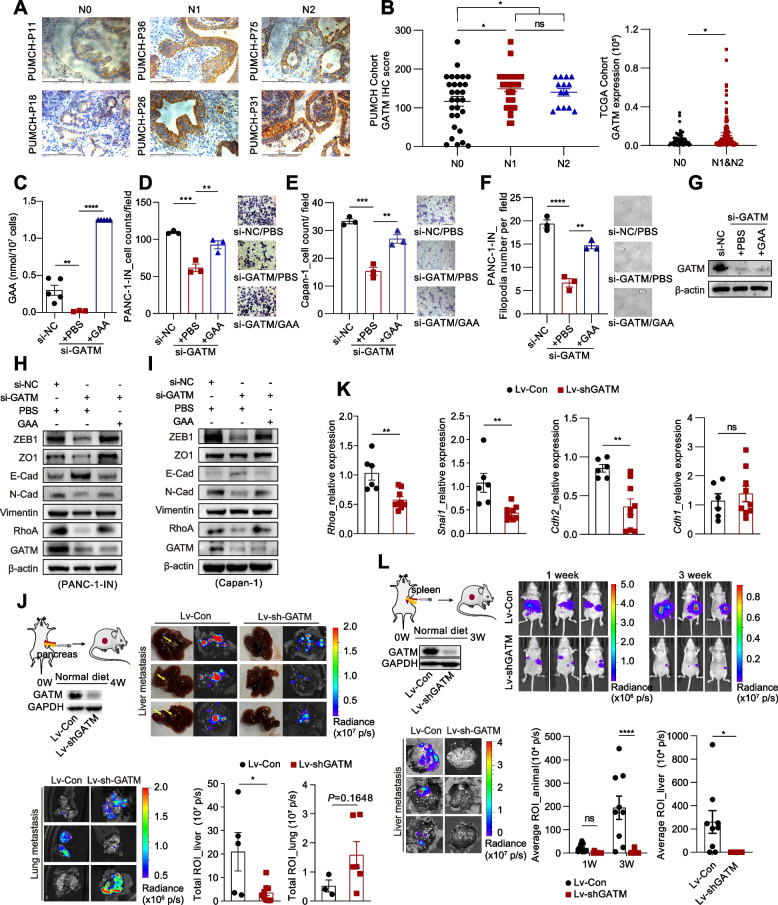
Knockdown of GATM suppressed liver metastasis of PDAC. **A** Representative images showing GATM IHC staining results in different PDAC specimens from PUMCH cohort. 100 × magnifying power. **B** The levels of GATM expression in pancreatic cancer with different N classification in PUMCH PDAC cohort (left) and TCGA PAAD cohort (right). **C** Quantitative measurement of intracellular GAA in PANC-1-IN cells by LC–MS/MS. The upper limit of GAA detection per 10^7^ cells was 1.25 nmol. (**D**-**E**) Transwell migration assay of pancreatic cancer cells with GATM knockdown or exogenous GAA (1 Mm) treatment. 200 × magnifying power. **F** Representative images showing the filopodia of PANC-1N cells after intervention. 400 × magnifying power. **G**-**I** Western blot showing β-actin and EMT marker proteins expression in pancreatic cancer cells. **J** Representative bright-field images, IVIS bioluminescence images and quantification of the liver and lung metastasis. NPG mice model. (K) RT-qPCR showing the EMT marker genes expression in primary tumors. **L** Representative IVIS bioluminescence images and quantification of liver metastasis in spleen injection mouse model. Data in (**B**) left and (**C**-**F**) are presented as mean ± SEM by ordinary one-way ANOVA (Dunnett’s multiple comparisons test). Data in (**B**) right and (**J**, **K**-**L**) are presented as mean ± SEM by unpaired t test. * *P* < 0.05, ** *P* < 0.01, *** *P* < 0.001, **** *P* < 0.0001

GAA can be absorbed from the diet or synthesized through the enzymatic reaction mediated by GATM. However, few studies have explored the role of GATM during PDAC metastasis. Thus, we analysed the expression of GATM in eighty-one PDAC specimens collected from Peking Union Medical College Hospital (PUMCH) (Table 1; Supplementary materials_0^6^). The immunohistochemical (IHC) staining results showed that the GATM expression level was higher in PDAC tumours with a lymph node metastasis stage of N1 or N2 than in N0-stage PDAC, consistent with the results of TCGA analysis (Fig. 3A-B). To fully evaluate the promoting role of GATM in PDAC metastasis, we chose pancreatic cancer cell lines with high GATM protein expression and knocked down the expression of GATM (Fig. S3A). After GATM knockdown, the intracellular GAA level was decreased and pancreatic cancer cell migration was significantly suppressed (Fig. 3C-E; Fig. S3B-D). GATM knockdown also significantly inhibited filopodia formation and cell–matrix adhesion in PANC-1-IN cells (Fig. 3F-G; S3F-H). Moreover, ZEB1, N-cadherin and RhoA were downregulated, and E-cadherin was upregulated after GATM knockdown (Fig. 3H-I; Fig. S3E). The inhibitory effect of GATM knockdown was attenuated after the addition of exogenous GAA (1 mM). However, we did not obtain definitive and consistent evidence that GATM could promote the proliferation of these pancreatic cancer cell lines (Fig. S3I-L).

**Table 1:** Clinicopathological features of 81 postoperative patients with pancreatic cancer

Patients’ characteristics	n	GATM^h^ (n, %) expression	Pearson χ^2^	*P*
**Sex**			1.035	0.439
Male	43	38(88.4)		
Female	38	36(94.7)		
**Age (years)**			0.297	0.586
≤ 60	34	16(47.1)		
> 60	47	25(53.2)		
**Location**			0.602	0.438
Head/neck	50	33(66.0)		
Body/tail	31	23(74.2)		
**Differentiation**			3.393	0.065
Low	28	5(17.8)		
Moderate/High	53	20(37.7)		
**Primary tumor size**			0.392	1.000
T1	13	0(0.0)		
T2 + T3	68	2(2.9)		
**Lymph node metastasis**			12.415	0.001
N0	29	21(72.4)		
N1 + N2	52	51(98.1)		
**TNM stage**			13.113	0.001
I	22	15(68.2)		
II + III	59	57(96.6)		

*GATM*^*h*^ GATM high expression

We next validated the role of GATM in promoting the liver metastasis of pancreatic cancer in vivo. The mice bearing orthotopic pancreatic tumours expressing higher levels of GATM developed more liver metastases, but there was no difference in lung metastasis between the groups (Fig. 3J; Fig. S3M). Tumours derived from GATM-knockdown cells expressed lower levels of *Snai1, Cdh2* and *Rhoa* than tumours-derived from controls (Fig. 3K). Since the primary tumours in the GATM-knockdown group weighed less than those in the control group (Fig. S3N), to exclude the impact of the reduction in primary tumours size on the number of cells available for metastasis formation, we further established a splenic injection model, in which the injected pancreatic cancer cells reached the liver directly to form metastases without first forming primary tumours. We observed that the mice in the GATM-knockdown group developed significantly fewer liver metastases than those in the control group (Fig. 3L).

### Three-dimensional epigenome reprogramming upregulates GATM, promoting PDAC metastasis

The highly metastatic pancreatic cancer cells (PANC-1-IN) and liver metastatic cancer cells (Capan-1) had higher levels of *Gatm* expression than the primary pancreatic cancer PANC-1 cells (Fig. S4A). To investigate the mechanism of *Gatm* upregulation, we first investigated the mutation and copy number of *Gatm* in TCGA PAAD cohorts and found no alterations (Fig. S4B). All five pancreatic cancer cell lines had moderate *Gatm* gene promoter methylation (Fig. S4C). However, no difference in *Gatm* promoter methylation was found between PDAC patients with different tumour stages (M0 vs. M1; N0 vs. N1) (Fig. S4D-E).

**Fig. 4:**
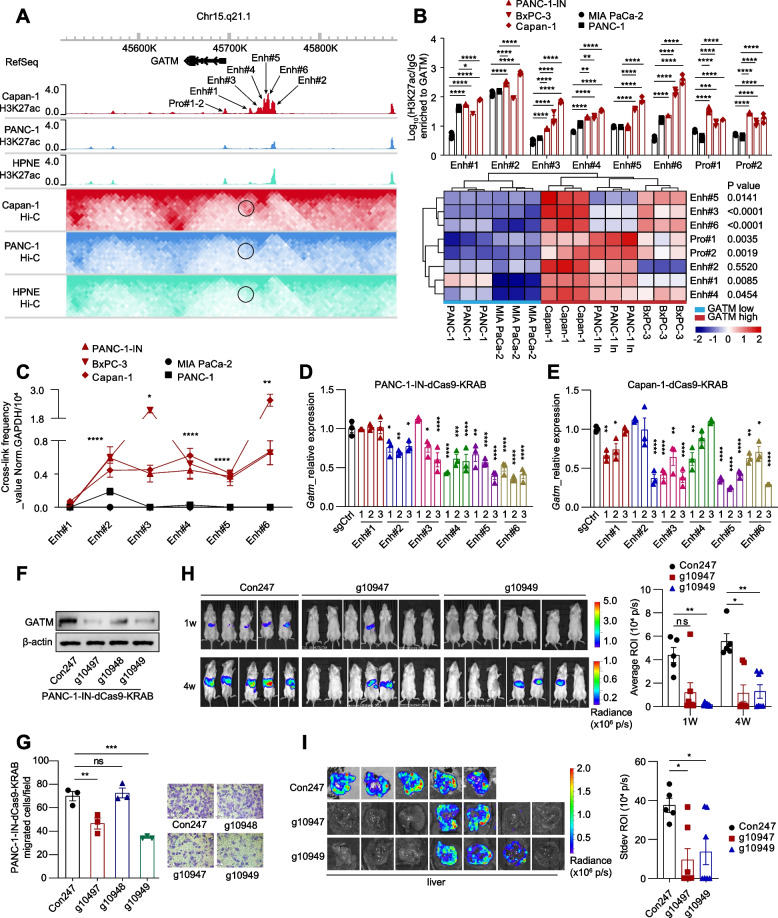
Three-dimensional epigenome reprogramming upregulates GATM expression promoting PDAC metastasis. **A** In situ Hi-C maps and histone ChIP-seq tracks surrounding the *Gatm* gene locus. The enhancer-promoter loop was showed by black circle. **B** ChIP-qPCR showing H3K27ac enriched to *Gatm* promoter and candidate enhancers. Heatmap visualization of ChIP-qPCR data that was divided into GATM low expression group and high expression group. **C** 3C-PCR showing the interaction between the candidate enhancers and *Gatm* promoter. **D**-**E** RT-qPCR showing *Gatm* expression in PANC-1-IN-dCas9-KRAB cells and Capan-1-dCas9-KRAB cells which were transfected by phU6 control and vectors carrying specific sgRNAs. (**F**-**G**) Western blot and transwell migration assay showing GATM expression and migration ability of PANC-1-IN-dCas9-KRAB cells. 200 × magnifying power. The groups named g10947, g10948 and g10949 stably expressed sgRNAs targeting Enh#4, 5 and 6 respectively. **H**-**I** Representative IVIS bioluminescence images and quantification of PDAC liver metastasis in splenic injection NPG mouse model. (h) animal level images; (i) dissected liver images. Data in (**B**-**C**), (**D**-**E**) and (**G**-**I**) are presented as mean ± SEM by 2way ANOVA (Tukey’s multiple comparisons test), unpaired t tests and ordinary one-way ANOVA (Tukey’s multiple comparisons test) respectively. * *P* < 0.05, ** *P* < 0.01, *** *P* < 0.001, **** *P* < 0.0001

We further performed integrated analysis of this *Gatm* expression data and the Hi-C and H3K27ac ChIP-seq data from our previous study [8]. The *Gatm* promoter and the candidate enhancer, located approximately 50 kb downstream of the promoter region, harboured more activating histone marks (H3K27ac) and had a stronger interaction in Capan-1 cells than in PANC-1 cells (Fig. 4A). Analysis of public data also showed that the *Gatm* promoter harboured more H3K27ac in the liver metastatic PDAC cell lines (Capan-1 and CFPAC-1) than in the primary pancreatic cancer cell line, MIA PaCa-2 (Fig. S4F). Moreover, a potential enhancer region was found at the same location in CFPAC-1 cells. Thus, the enhancer-promoter interaction might cause upregulation of GATM.

To validate this epigenome-metabolome mechanism, we first performed ChIP‒qPCR. H3K27ac enrichment at the *Gatm* promoter and the enhancer region was significantly higher in pancreatic cancer cells with high *Gatm* expression than in those with low *Gatm* expression (Fig. 4B). We next performed chromosome conformation capture-quantitative PCR (3C-qPCR) and detected strong interactions between the *Gatm* promoter and the enhancer in cells with high *Gatm* expression (Fig. 4C). Furthermore, we performed CRISPR interference (CRISPRi) via the dCas9-KRAB system to precisely silence the candidate enhancer by designing three guide RNAs (gRNAs) targeting each site (Fig. S4G). Transfection of the gRNAs targeting the candidate enhancer resulted in significant downregulation of GATM expression in pancreatic cancer cells (Fig. 4D-E). Moreover, we combined multiple gRNAs (targeting Enh#4, 5, and 6), and their transfection led to a significant silencing effect, but this effect was not better than that of any gRNA alone (Fig. 4F; Fig. S4H). Targeting Enh#4 by g10947 and Enh#6 by g10949 was then confirmed to significantly suppress the migration and pseudopodia formation of pancreatic cancer cells (Fig. 4G; Fig. S4I). In vivo, g10947- and g10949-mediated GATM downregulation effectively suppressed primary tumour growth in a BALB/c nude mouse model (Fig. S4J). In the PDAC metastasis model established in NPG mice by splenic injection, g10947- and g10949-mediated GATM downregulation significantly blocked the formation of liver metastases (Fig. 4H-I). These data revealed that 3D epigenome reprogramming led to GATM upregulation in some pancreatic cancer cells, indicating the high aggressiveness and metastatic potential of the affected cells.

### GAA metabolism promotes HMGA-induced EMT via MYC upregulation

GAA is the immediate precursor of creatine biosynthesis. The creatine kinase B (CKB)-dependent creatine-phosphagen system was reported to facilitate the invasiveness of pancreatic cancer cells [27]. To determine whether GAA drives PDAC metastasis through creatine-mediated mechanisms, targeted metabolomics analysis of pancreatic cancer cells was performed (Fig. S5A; Supplementary materials_0^4^). Significantly differential metabolites were classified into three clusters (Fig. S5B). The metabolites in Cluster 1, including GAA, were positively regulated by high GATM expression and exogenous GAA treatment; those in Cluster 2 were negatively regulated by exogenous GAA treatment; and those in Cluster 3 were negatively regulated by high GATM expression. GATM knockdown led to accumulation of upstream metabolites, which were collectively referred to as Cluster 3. Arginine biosynthesis was the most significantly enriched pathway (Fig. S5C). However, the levels of metabolites in the creatine-phosphagen system, categorized into Cluster 2, did not change after GATM knockdown (Fig. S5D-G). In in vivo experiments, the creatine level was not decreased in tumours derived from cells with GATM knockdown compared to those derived from control cells (Fig. S5H). No changes in the plasma levels of creatine and phosphocreatine were observed in mice receiving the GAA-containing diet compared to control mice (Fig. S5I-J).

**Fig. 5:**
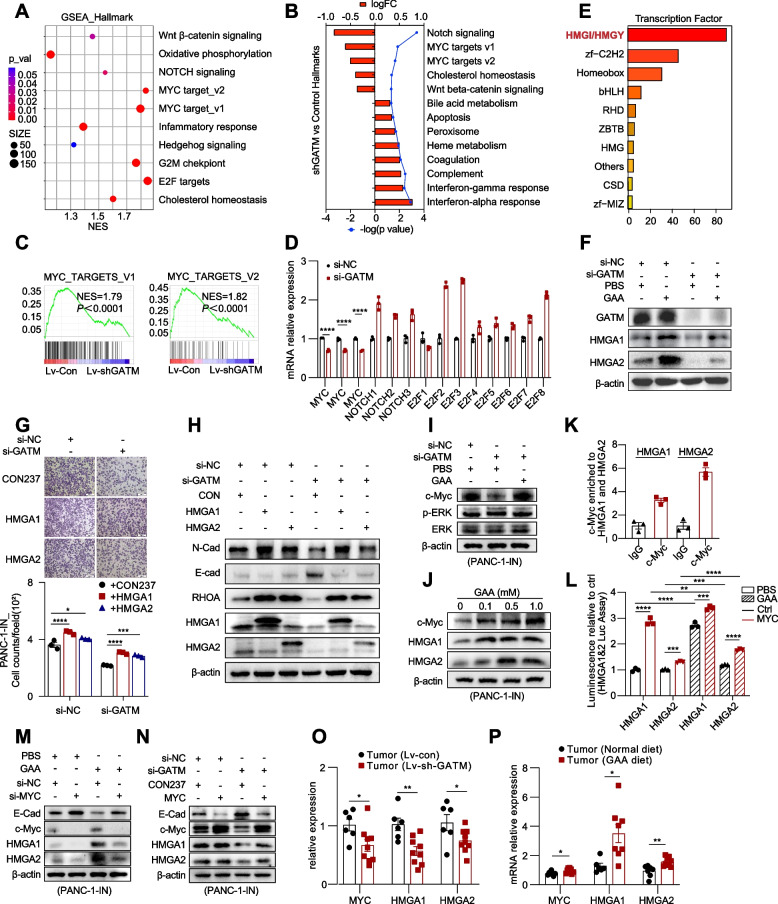
GAA metabolism promotes HMGA-induced EMT via upregulated MYC expression. **A**-**B** GSEA (left) and GSVA (right) results of the hallmark pathways based on DEGs between PANC-1-IN-Lv-Con and PANC-1-IN-Lv-sh-GATM. **C** GSEA result of MYC target pathway based on DEGs. **D** RT-qPCR showing the expression of *Myc*, *Notch* and *E2f* after GATM knockdown. **E** Identification of transcription factor proteins family based on differentially expressed transcription factor genes. **F** Western blot showing HMGA1 and HMGA2 expression after GATM knockdown. **G**-**H** Migration ability analysis and western blot showing EMT marker proteins expression of pancreatic cancer cells after GATM knockdown and HMGA1/2 overexpression. **I**-**J** Western blot showing the expression of c-Myc, HMGA1, HMGA2, and ERK/p-ERK in pancreatic cancer cells after intervention of GAA metabolism. **K** CUT&RUN analysis showing levels of c-Myc enriched to *Hmga1* and *Hmga2* promoters. **L** Luciferase reporter gene assay showing c-Myc protein targeting the promoter of Hmga1 and Hmga2. **M**–**N** Western blot showing the expression of c-Myc-HMGA-E-cadherin in PANC-1-IN cells. **O**-**P** RT-qPCR showing the expression of *Myc*, *Hmga1* and *Hmga2* in xenograft tumors. Data in (G, L) and (D, O,-P) are presented as mean ± SEM by ordinary one-way ANOVA (Tukey’s multiple comparisons test) and unpaired t test respectively. * *P* < 0.05, ** *P* < 0.01, *** *P* < 0.001, **** *P* < 0.0001

To investigate the mechanism by which GAA metabolism promotes PDAC metastasis, RNA-seq analysis was performed to compare the transcriptomes of pancreatic cancer cells with GATM knockdown and control cells was performed (Fig. S6A; Supplementary materials_0^5^). The DEGs were enriched mainly in metastasis-related pathways [28, 29] (Fig. S6B). The intersection of the DEGs from the three RNA-seq datasets contained upregulated genes (UPs) and downregulated genes (DWs) (Fig. S6C). DWs were expressed at significantly lower levels in liver metastases than in primary tumours (Fig. S6D-E). Kaplan‒Meier analysis revealed that high expression of UPs and low expression of DWs were significantly associated with poor prognosis in PDAC patients (Fig. S6D-E). GSEA and gene set variation analysis (GSVA) showed that the MYC target pathway was one of the most differentially enriched pathways (Fig. 5A-C). RT‒qPCR further verified the decreased expression of the *Myc* gene after GATM knockdown (Fig. 5D). However, the expression levels of *Notch* and *E2f2F* did not decrease and even increased to some degree. High expression of MYC has been reported to regulate the metastatic ability of PDAC [30].

We next predicted the differentially expressed transcription factor genes and identified their corresponding protein families. The high mobility group I/Y proteins (HMGI/HMGY) family ranked first (Fig. 5E). In the new terminology, the HMGI/HMGY family is referred to as the HMGA family, and its members contain a highly conserved DNA-binding domain called the ‘AT hook’. Aberrant expression of HMGA proteins is associated with many cancers, including PDAC [31]. Here, high expression of HMGA1 and HMGA2, two important members of the HMGI/HMGY family, was significantly associated with a poor prognosis in patients with pancreatic cancer (Fig. S6F). HMGA proteins play essential roles during pancreatic cancer metastasis [32–34]. Downregulated expression of HMGA1 and HMGA2 was observed after GATM knockdown, and exogenous GAA (1 mM) treatment rescued their expression (Fig. 5F; Fig. S6G). After overexpression of HMGA1 and HMGA2, the suppression of PANC-1-IN cell migration and EMT was significantly mitigated (Fig. 5G-H).

Many studies have reported that c-Myc targets the promoters of *HMGA1* and *HMGA2*, transcriptionally enhancing their expression in various types of cancer, including PDAC [35–40]*.* Elevated expression of HMGA1 was found to require the presence of oncogenic Ras in colon cancer [41]. However, we observed a significant decrease in c-Myc expression after GATM knockdown, but no change in the p-ERK and ERK protein levels (Fig. 5I; Fig. S6I). In vitro, as the GAA concentration increased, the expression levels of c-Myc, HMGA1 and HMGA2 increased noticeably (Fig. 5J; Fig. S6H). Thus, we speculated that GAA might promote the migration of pancreatic cancer cells through c-Myc-mediated HMGA protein expression. We then performed CUT&RUN and found significant enrichment of the c-Myc protein on the *HMGA1* and *HMGA2* promoters (Fig. 5K). ChIP-seq data from HCT-116 and 293 T cells also showed that c-Myc was clearly enriched in the promoter regions of *HMGA1* and *HMGA2* (Fig. S6J). The luciferase reporter assay results verified *HMGA1* and *HMGA2* as target genes of c-Myc, and GAA treatment significantly enhanced the luminescence signal (Fig. 5L). We next knocked down c-Myc in pancreatic cancer cells, which blocked the GAA-induced expression of HMGA1 and HMGA2 (Fig. 5M; Fig. S6K). Furthermore, c-Myc overexpression in pancreatic cancer cells with GATM knockdown restored HMGA1 and HMGA2 expression (Fig. 5N; Fig. S6K). In xenograft tumours, GATM knockdown suppressed the expression of *MYC*, *HMGA1*, and *HMGA2*, and GAA diet feeding stimulated their expression (Fig. 5O-P). These results suggest that GAA could promote pancreatic cancer cell migration and EMT through c-Myc-mediated expression of the HMGA1 and HMGA2 proteins.

### GAA promotes transcription-activating histone modifications at metastasis-related genes

**Fig. 6:**
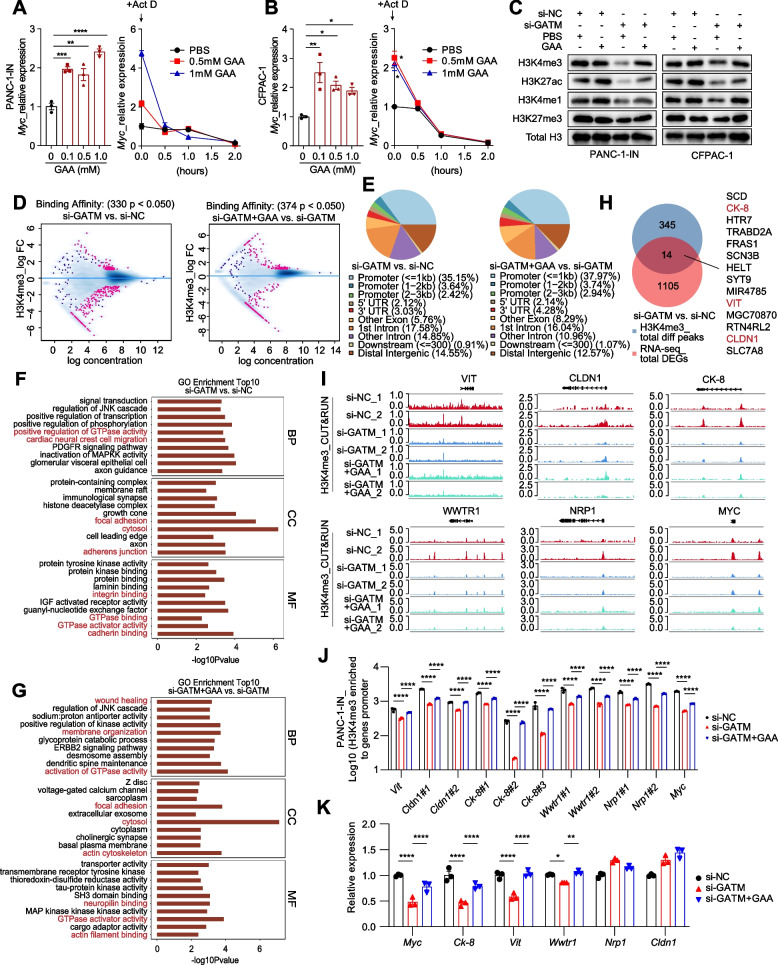
GAA promotes transcriptionally active histone modifications at metastasis related genes. **A**-**B** RT-qPCR showing *Myc* RNA degradation rate after exogenous GAA treatment. Actinomycin D (Act D) was used to inhibit the DNA-dependent RNA polymerase activity. **C** Western blot showing the expression of active and repressive histone modifications at the cellular level. **D** The MA plot showing concentration of differential H3K4me3 peak between si-GATM and si-NC or si-GATM + GAA and si-GATM. The degree of color represents the *P* value. **E** The annotated distribution of differential H3K4me3 peaks on the gene functional elements. **F**-**G** The GO enrichment analysis of differential H3K4me3 peaks-annotated genes. **H** The Venn analysis of differential expressed genes and differential peaks-related genes. **I** Histone CUT&RUN tracks showing the enrichment peaks of H3K4me3 at metastasis-related genes*.* **J** ChIP-qPCR showing the level of H3K4me3 enriched to the promoters of identified metastasis-related genes and *Myc*, normalized to IgG group. **K** RT-qPCR showing the mRNA expression of metastasis-related genes. Data in (A-B, J-K) are presented as mean ± SEM by ordinary one-way ANOVA (Tukey’s multiple comparisons test). * *P* < 0.05, ** *P* < 0.01, *** *P* < 0.001, **** *P* < 0.0001

Many studies have reported the nonmetabolic functions of metabolic enzymes in malignant tumours; for example, these enzymes orchestrate histone modifications during PDAC metastasis [6, 42]. H3K4me3 and H3K27ac are recognized as histone markers of active promoters and enhancers and regulate the expression of metastasis-associated genes [43, 44]. However, whether GAA metabolism impacts histone modifications in pancreatic cancer cells is unknown. The mRNA expression of *Myc* gene was upregulated as the GAA concentration increased, without attenuation of RNA degradation (Fig. 6A-B). Thus, we speculated that GAA might promote the expression of metastasis-related genes by affecting histone modifications. Indeed, we found that GAA promoted activating modifications (H3K4me3, H3K27ac, and H3K4me1) at the cellular level but had no noticeable impact on the level of the repressive modification H3K27me3 (Fig. 6C).

To investigate the genome-wide changes in H3K4me3 and H3K27ac, we performed CUT&RUN analysis in pancreatic cancer cells after GATM knockdown and GAA rescue treatment. The concentration of differential H3K4me3 peaks decreased significantly after GATM knockdown (Fig. 6D). The differential of H3K4me3 peaks were mainly distributed in promoter regions (Fig. 6E). The results of GO enrichment analysis of genes annotated to contain differential peaks showed that metastasis-related pathways were significantly differentially enriched between si-GATM and si-NC cells and between si-GATM + GAA and si-GATM cells (Fig. 6F-G) (Supplementary materials_0^5^). We further identified 14 DEGs by integrated analysis of CUT&RUN data and RNA-seq data of PANC-1-IN-si-NC and PANC-1-IN-si-GATM cells (Fig. 6H). *Vitrin* (*Vit*), *cytokeratin-8* (*Ck-8* or *Krt8*) and *claudin1* (*Cldn1*) are associated with cell adhesion and migration and participate in cancer metastasis [45, 46]. These three genes had lower levels of H3K4me3 enrichment after GATM knockdown (Fig. 6I). In addition, H3K4me3 enrichment was also decreased at the promoters of *Myc*, *WW domain-containing transcription regulator protein 1 *(*Wwtr1)* and *neuropilin 1* (*Nrp1),* which participate in cancer metastasis, after GATM knockdown (Fig. 6I). Although a perfect rescue effect of GAA was not observed in the track plot, we soundly validated the results through ChIP‒qPCR and RT‒qPCR (Fig. 6J). The mRNA expression levels of *Myc, Ck-8, Vit*, and *Wwtr1* were decreased after GATM knockdown and were restored by GAA treatment (Fig. 6K).

In addition, H3K27ac modification also showed significant alterations after intervention with GAA metabolism (Fig. S7A-E). However, the most prominently changed pathways were mainly related to autophagy, cell division, and DNA damage (Fig. S7F-G). Integrated analysis of CUT&RUN and RNA-seq data showed that GATM knockdown resulted in the downregulation of *Nrp1* accompanied by decreased H3K27ac modification levels (Fig. S7H), which was validated by ChIP-qPCR (Fig. S7K). In addition, GATM Knockdown led to the upregulation of 20 genes by increasing their H3K27ac modification levels (Fig. S7F-G). These 20 genes were enriched mainly in the apoptosis pathway (Fig. S7J).

### GAA promotes H3K4me3 of metastasis-related genes by upregulating histone methyltransferases

**Fig. 7:**
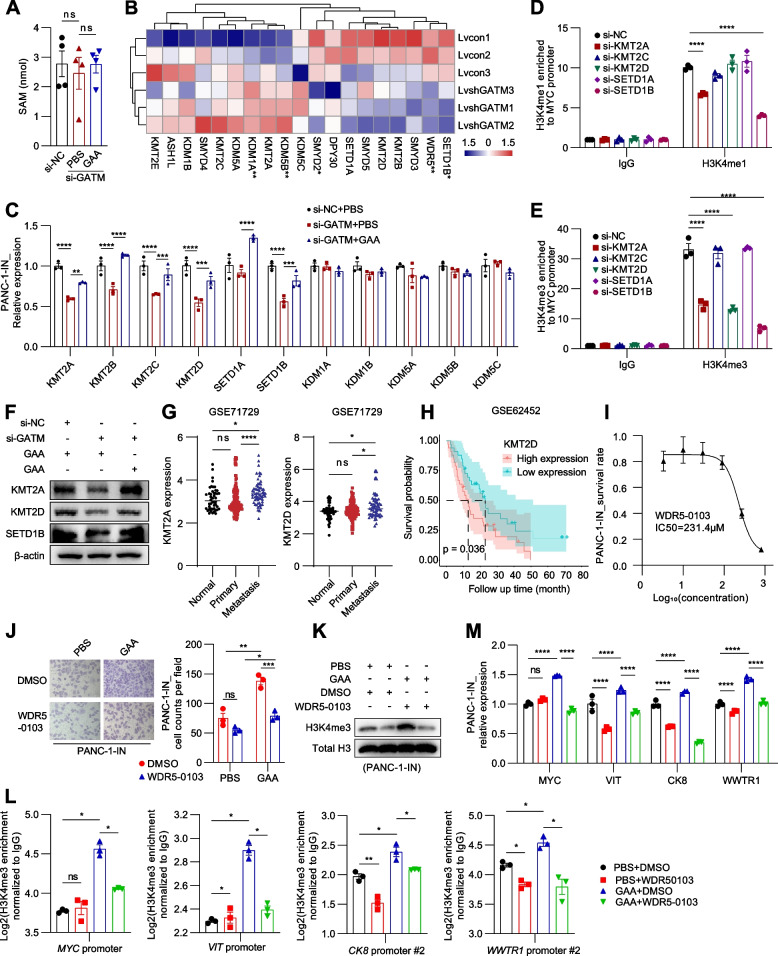
GAA promotes H3K4me3 of metastasis-related genes by upregulating histone methyltransferases expression. **A** Quantitative measurement of SAM in PANC-1-IN cells by LC–MS/MS. **B** The expression Heatmap of HMTs and HDMs. The color degree represents the relative expression level. **C** RT-qPCR showing the expression of different methyltransferases and demethylases of H3K4 after GATM knockdown and exogenous GAA (1 mM) treatment. **D**-**E** CUT&RUN experiment showing levels of H3K4me3 and H3K4me1 enriched to *Myc* promoter after knockdown of different methyltransferases of H3K4. **F** Western blot showing the expression of HMTs in PANC-1-IN cells. **G** GSE71729 data analysis showing the levels of epigenetic enzymes expression among normal pancreas, primary PDAC and metastasis of PDAC. **H** Kaplan–Meier survival analysis by using the median cutoff value of KMT2D expression by using GSE62452 dataset. **I** The IC50 curve of WDR5-0103 in PANC-1-IN cells. **J** Transwell migration assay of pancreatic cancer cells after intervention. **K** Western blot showing the expression of H3K4me3 after GAA and WDR5-0103 treatment. **L** CUT&RUN analysis showing the H3K4me3 enriched to the promoters of metastasis-related genes. **M** RT-qPCR showing the mRNA expression of metastasis-related genes. Data in (A, C-E, J, L-M) are presented as mean ± SEM by ordinary one-way ANOVA (Tukey’s multiple comparisons test). * *P* < 0.05, ** *P* < 0.01, *** *P* < 0.001, **** *P* < 0.0001

To investigate the mechanism by which GAA metabolism regulates H3K4me3, we first measured the level of S-adenosyl methionine (SAM), the methyl-donating substrate of histone methyltransferases (HMTs). However, we did not find any change in the SAM level in pancreatic cancer cells after disruption of GAA metabolism (Fig. 7A). Through analysis of RNA-seq data of PANC-1-IN cells after GATM knockdown, we observed a decreasing trend in HMT expressions and an increasing trend in histone demethylase (HDM) expressions (Fig. 7B). We then measured the expression of HMTs and HDMs by RT‒qPCR. The HMT expression levels changed significantly, while no changes in HDM expression were observed (Fig. 7C). Furthermore, lysine methyltransferase 2A (KMT2A), lysine methyltransferase 2D (KMT2D) and SET domain containing 1B (SETD1B) were found to induce the depletion of H3K4me3 and H3K4me1 on the *Myc* gene promoter (Fig. 7D-F). KMT2A and KMT2D had higher levels of expression in metastases than in primary tumours and normal pancreatic tissues (Fig. 7G). High expression of KMT2D was related to poor survival outcomes of pancreatic cancer patients (Fig. 7H).

WD repeat domain 5 (WDR5) is an adaptor protein and is essential for the enzyme activity of all H3K4me3-specific HMTs [47]. Therefore, we further inhibited WDR5 with WDR5-0103 to suppress H3K4me3 modification in pancreatic cancer cells. A concentration equal to one-half of the IC50 value was used (Fig. 7I). WDR5-0103 significantly blocked GAA-induced migration of pancreatic cancer cells (Fig. 7J). As expected, WDR5-0103 reduced the expression of H3K4me3 at the cellular level (Fig. 7K). Next, we detected H3K4me3 enrichment on metastasis-related genes. Treatment with WDR5-0103 significantly blocked the GAA synthesis-promoting H3K4me3 modifications at *Myc*, *Vit*, *Ck-8* and *Wwtr1* in PANC-1-IN cells (Fig. 7M). Correspondingly, the expression of *Myc*, *Vit*, *Ck-8* and *Wwtr1* was suppressed by WDR5-0103 (Fig. 7L). The same results were obtained in another pancreatic cancer cell line (Fig. S8 A-F).

In addition, we measured the level of acetyl-CoA in pancreatic cancer cells but did not observe a significant change after GATM knockdown or GAA treatment (Fig. S9A). However, histone acetyltransferase expression changed substantially (Fig. S9B-C). Several enhancer regions were determined to loop to the promoter of *Myc* in pancreatic cancer cells [48, 49] (Fig. S9D-E). We then validated the *Myc* promoter and enhancer regions by ChIP‒qPCR (Fig. S9F-I). Bromodomain-containing 4 (BRD4) has been reported to bind to enhancers containing H3K27ac modifications and the promoter regions of *Myc* [50, 51]. Thus, we used JQ1 (3 μM) to target BRD4-mediated transcriptional regulation and found that this treatment significantly blocked GAA-mediated *Myc* transcription (Fig. S9J-K). These results revealed that GAA could promote H3K4me3 and H3K27ac deposition by regulating epigenetic enzyme expression during PDAC metastasis.

## Discussion

Metabolites involved in arginine and proline metabolic pathways, such as arginine, polyamines, and creatine, have been shown to support tumour growth or metastasis [25, 27, 52–56]. Arginine deprivation is emerging as a targeted therapeutic approach for arginine-auxotrophic cancers including pancreatic cancer [25, 57, 58]. We found that arginine depletion hindered the migration of pancreatic cancer cells with low ASS1 expression, and that the migration of these cells could be restored by GAA supplementation. In humans, GAA is produced mainly in the kidney. After its synthesis, GAA circulates to the liver and pancreas, functioning as a crucial amino acid derivative [26]. Dietary GAA has been found to enhance muscular performance in healthy individuals and prevent muscle mass loss in patients with chronic renal failure [59]. In our study, either GAA supplementation or high expression of GATM promoted PDAC cell migration in vitro and PDAC liver metastasis in vivo. Since some of the orthotopic models contained fewer circulating tumour cells, which could lead to differences in the liver metastasis rate, we directly injected pancreatic cancer cells into the spleen. However, we still cannot completely separate proliferation defects from metastatic seeding defects. Further studies are needed to distinguish the direct effects of GAA on tumour growth and distant metastasis.

We found that GAA promoted PDAC liver metastasis but did not influence lung metastasis. Anatomically, pancreatic venous blood carrying disseminating pancreatic cancer cells flows into the liver through the portal vein, leading to a high rate of liver metastasis. We observed that the liver was the most common site of metastasis, with liver metastasis accounting for 34% of all postoperative metastases (Fig. S3O). Additionally, Schild et al*.* postulated that unique metabolic adaptations were required for organ-specific metastatic colonization [60]. The Human Metabolome Database (HMDB) indicates that GAA is physiologically deposited in the brain, kidney, liver, and placenta. Thus, we speculated that GAA concentration is higher in the liver, creating more favourable conditions for the colonization of spreading PDAC cells. Indeed, we found that the liver had a higher GAA concentration than the pancreas or lungs in mice (Fig. S3P). Many studies have reported the overexpression of GAA transporters in liver metastatic tumours compared to primary tumours in diverse cancers, including pancreatic cancer [56, 61–63]. These findings suggest the potential metabolic dependency of PDAC liver metastasis on GAA utilization. Moreover, one study reported that highly liver-metastatic cancer cells contained greater amounts of intracellular arginine and displayed a higher IC_50_ of arginase [64]. As the liver is a major organ of arginase localization, it can generate an arginine- deprived environment surrounding metastatic cancer cells. However, metastatic pancreatic cancer cells with high ASS1 and GATM expression maintained higher levels of intracellular arginine and GAA than those with low ASS1 and GATM expression. This observation provides an alternative perspective to explain why GATM knockdown significantly suppressed the liver metastasis of pancreatic cancer.

Many studies have reported that enhancer reprogramming can promote cancer metastasis [44, 65]. For instance, Jae-Seok et al. demonstrated that FOXA1-dependent enhancer reprogramming activated a transcriptional program in embryonic foregut endoderm and induced pancreatic cancer metastasis [44]. In this study, we found that the active enhancer looped to the *Gatm* gene, contributing to upregulated GATM expression and an increase in intracellular GAA. However, we did not exclude the impact of liver environmental features, such as hypoxia, secreted cytokines or hepatocytes, which might also contribute to epigenetic reprogramming, or other unknown mechanisms leading to the enhancement of GAA biosynthesis. Despite this limitation of our study, enhancers newly emerging or aberrantly activated during cancer progression have been increasingly considered as a focus of therapeutic targeting [66].

High expression of GATM was found to promote liver metastasis via creatine-mediated EMT in colorectal cancer [56]. However, we did not observe decreases in metabolites involved in the creatine-phosphagen system in pancreatic cancer cells after GATM knockdown or increases in those metabolites in the plasma of mice fed the GAA diet. In contrast, the intracellular creatine level decreased after GAA treatment. Moreover, GAA treatment did not affect the expression of GAMT, CKB and CKMT2, the key enzymes involved in creatine-phosphocreatine biosynthesis (Fig. S5K). However, SLC6A8 and SLC6A6 are important transporters of guanidino compounds, such as GAA and creatine [26, 67]. Thus, we speculated that increased GAA influx may competitively limit the crucial uptake of creatine and other guanidino compounds, an effect that does not change the creatine/phosphocreatine levels, and that GAA may play a more important role than creatine and phosphocreatine in PDAC liver metastasis (Fig. S5L).

Alterations in activating H3K4me3 marks (or the epigenetic enzymes) are tightly related to PDAC metastasis. For instance, PHD finger protein 13 (PHF13) induces increased deposition of the activating epigenetic marks H3K4me3 on genes critical to pancreatic cancer cell migration and invasion [68]. Hypoxia causes inactivation of lysine demethylase 5A (KDM5A), increasing the H3K4me3 level and regulating the expression of EMT-associated genes [43]. Here, we found that GAA promoted H3K4me3 modification of metastasis-related genes (*Vit. Ck-8,* and *Cldn1*) in pancreatic cancer cells. The proteins encoded by these genes might regulate PDAC metastatic phenotypes in cooperation with HMGA1 and HMGA2. In addition, GAA might play important roles in regulating apoptosis and oxidative stress in pancreatic cancer cells through H3K27ac reprogramming, a possibility that requires further studies. Finally, we elucidated that GAA promoted the transcription-activating histone modifications by regulating critical HMTs and HATs. However, the underlying mechanism by which GAA regulates various epigenetic enzymes is unclear.

## Conclusions

In conclusion, our findings revealed that enhanced GAA anabolism, induced by 3D epigenome reprogramming, could promote PDAC liver metastasis by promoting c-Myc-mediated HMGA protein expression and reprogramming transcription-activating histone modification, indicating a close and complicated relationship between two important hallmarks of cancer — metabolic remodelling and epigenetic reprogramming — during pancreatic cancer liver metastasis. In the future, the myriad altered metabolites interacting with epigenetic reprogramming during pancreatic cancer metastasis need to be identified. The development of new strategies targeting the epigenome-metabolome interaction program might help to combat distant metastasis and improve the survival outcomes of patients with pancreatic cancer.

## Supplementary Information

**Additional file 1.**

**Additional file 2.**

**Additional file 3.**

**Additional file 4.**

**Additional file 5.**

**Additional file 6.**

**Additional file 7.**

**Additional file 8.**

**Additional file 9: Fig. S1. **A high level of intracellular GAA wasassociated with PDAC liver metastasis. **Fig. S2. **GAA promoted liver metastasis of PDAC. **Fig. S3. **Knockdown of GATM suppressed livermetastasis of PDAC. **Fig. S4. **Three-dimensional epigenomereprogramming upregulates GATM expression promoting PDAC metastasis. **Fig. S5. **Metabolic alteration after GAAmetabolism disturbance. **Fig. S6. **GAA metabolism promotes HMGA-induced EMTvia upregulated MYC expression. **Fig. S7. **GAA promotes H3K27ac modifications atcell cycle and apoptosis-related genes. **Fig. S8. **GAA promotes H3K4me3 ofmetastasis-related genes by upregulating histone methyltransferases expression. **Fig. S9. **GAA metabolism promotes H3K27acmodification at the enhancers of the *MYC* gene.

## Data Availability

All data generated or analysed in this study are included in this published article and its supplementary information files.

## Abbreviations

PDAC: Pancreatic ductal adenocarcinoma
GATM: Glycine amidinotransferase
GAA: Guanidinoacetic acid
SAM: S-adenosyl methionine
HMGA: High mobility group AT-Hook
3D: Three-dimension
ChIP: Chromatin immunoprecipitation
3C: Chromatin capture conformation quantitative
ASS1: Argininosuccinate synthetase 1
EMT: Epithelial-mesenchymal transition
ODC1: Ornithine decarboxylase 1
DEG: Differential expressed genes
SLC6A8/6/13: Solute carrier family 6 member 8/6/13
NPG: NOD. Cg-Prkdc^scid^ Il2rg^tm1Vst^/Vst
H3K27ac: Histone 3 lysine 27 acetylation
GSVA: Gene set variation analysis
GSEA: Gene set enrichment analysis
H3K4me3: Histone 3 lysine 4 trimethylation
H3K4me1: Histone 3 lysine 4 monomethylation
SEs: Superenhancers
BRD4: Bromodomain-containing 4
PUMCH: Peking Union Medical College Hospital
LIPC: Hepatic lipase C
FOXA1: Forkhead box A1
KDM6A: Lysine demethylase 6A
HGF: Hepatic growth factor
CKB: Creatine kinase B
HMTs: Histone methyltransferases
HATs: Histone acetyltransferases
KMT2A: Lysine methyltransferase 2A
KMT2D: Lysine methyltransferase 2D
SETD1B: SET domain containing 1B
VIT: Vitrin
CK-8: Cytokeratin 8
CLDN1: Claudin 1
WWTR1: WW domain -Containing Transcription regulator Protein 1
NRP1: Neuropilin 1
