# Supplementary material for: Epigenetic reprogramming-induced guanidinoacetic acid synthesis promotes pancreatic cancer metastasis and transcription-activating histone modifications
Source: J Exp Clin Cancer Res. 2023 Jun 28;42:155. doi: 10.1186/s13046-023-02698-x (PMC10304235; doi:10.1186/s13046-023-02698-x)
Supplement: Supplementary file 8 — Additional file 8. [file 13046_2023_2698_MOESM8_ESM.pptx]

## Slide 1
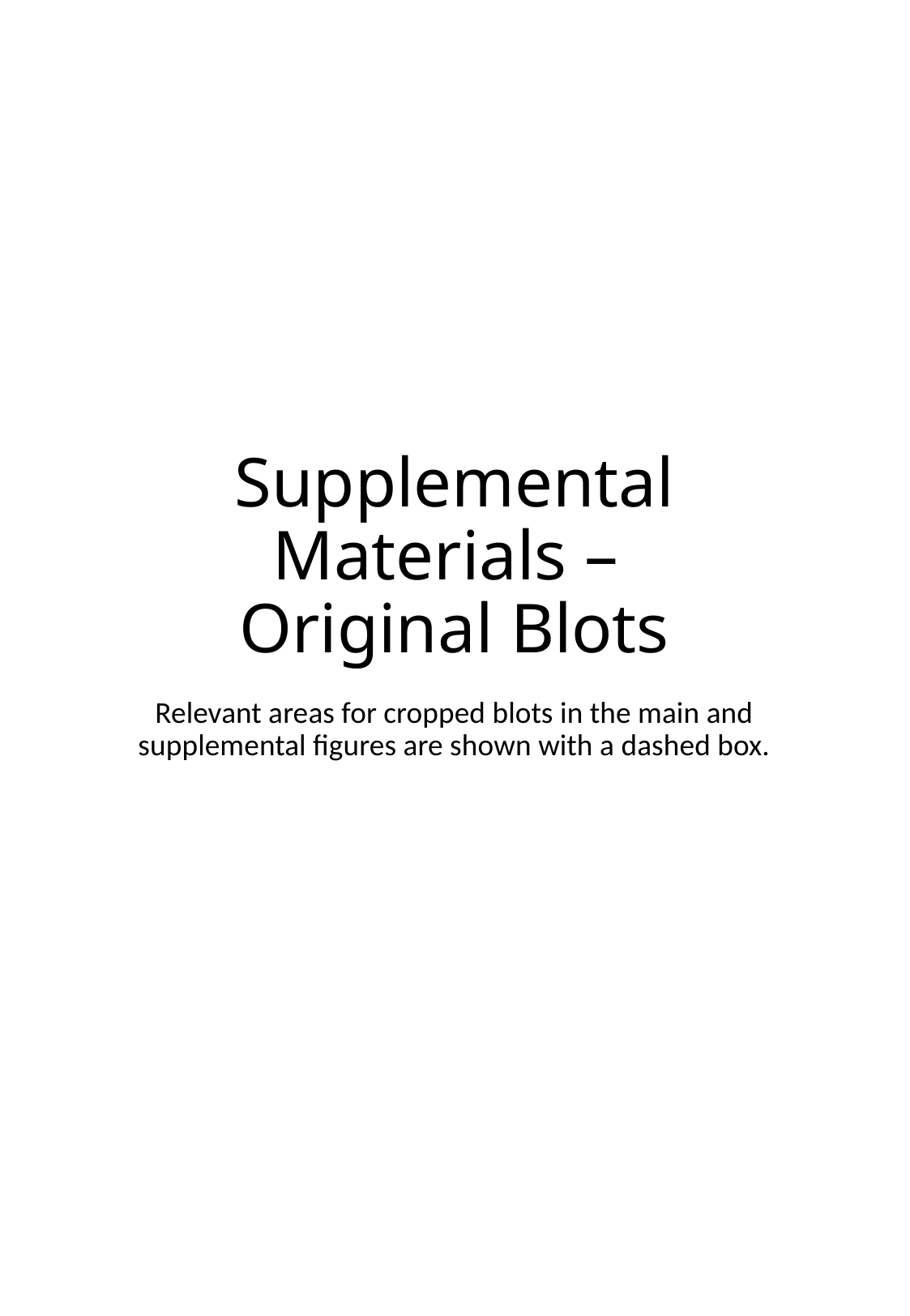

# Supplemental Materials – Original Blots
Relevant areas for cropped blots in the main and supplemental figures are shown with a dashed box.

## Slide 2
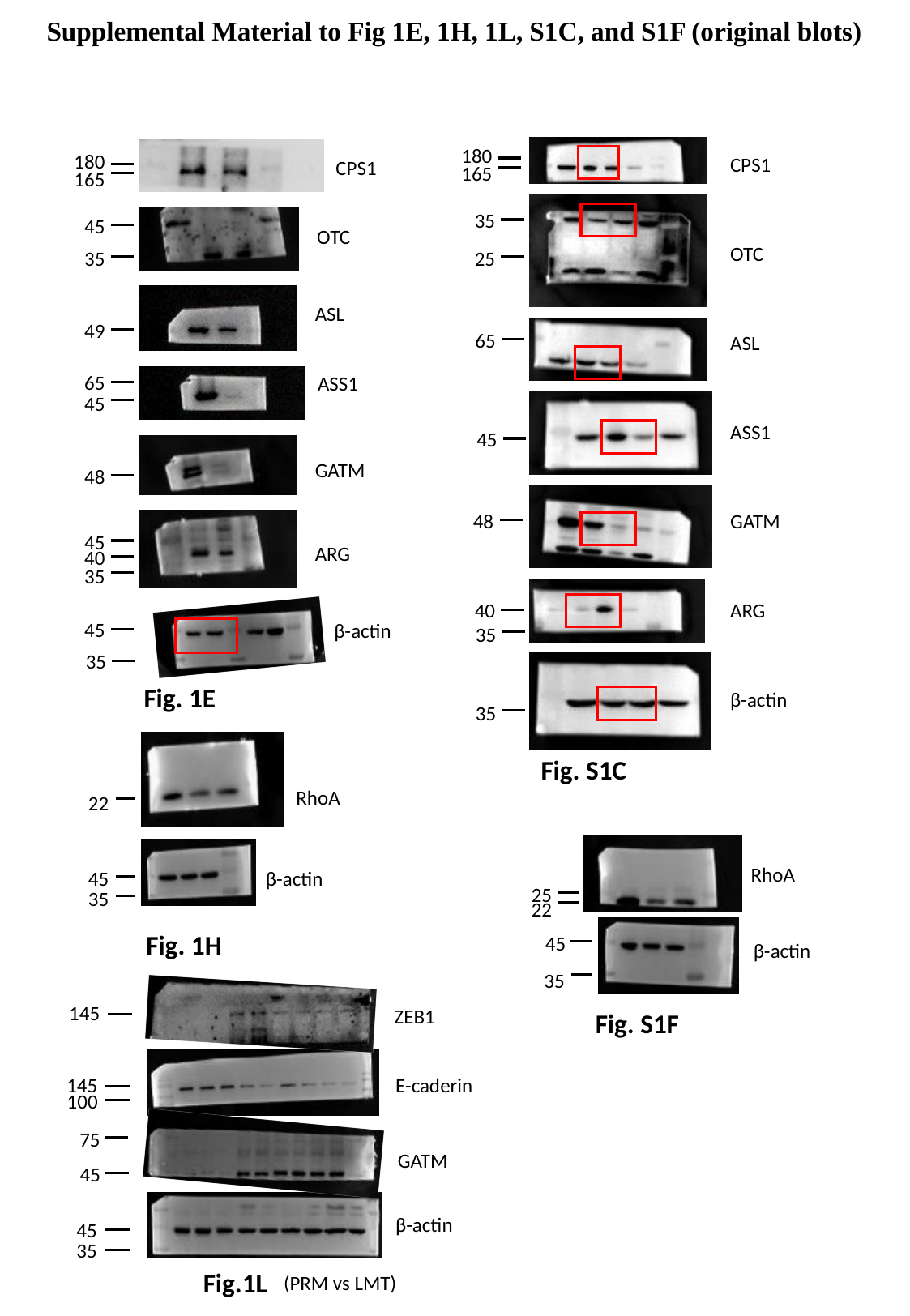

Supplemental Material to Fig 1E, 1H, 1L, S1C, and S1F (original blots)
180
CPS1
165
35
OTC
25
65
ASL
ASS1
45
48
GATM
40
ARG
35
β-actin
35
Fig. S1C
180
CPS1
165
45
OTC
35
ASL
49
65
ASS1
45
GATM
48
45
ARG
40
35
45
β-actin
Fig. 1E
35
RhoA
22
β-actin
45
35
Fig. 1H
RhoA
25
22
β-actin
35
Fig. S1F
45
145
ZEB1
145
E-caderin
100
GATM
β-actin
45
35
Fig.1L
(PRM vs LMT)
75
45

## Slide 3
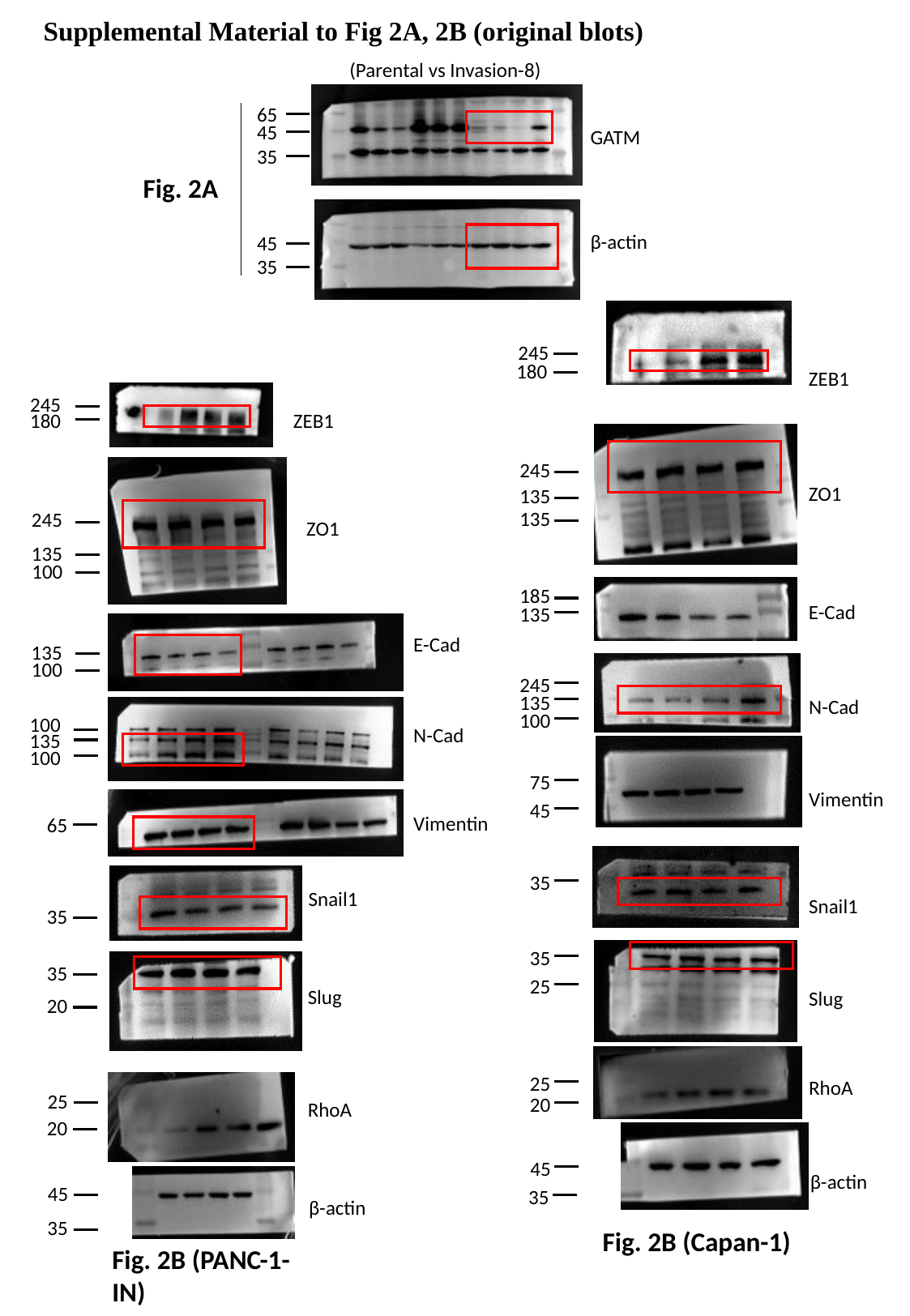

Supplemental Material to Fig 2A, 2B (original blots)
(Parental vs Invasion-8)
65
45
35
45
35
GATM
Fig. 2A
β-actin
245
180
ZEB1
245
ZO1
135
135
185
E-Cad
135
245
135
N-Cad
100
75
Vimentin
45
35
Snail1
35
25
Slug
25
RhoA
20
45
β-actin
35
Fig. 2B (Capan-1)
245
180
ZEB1
245
ZO1
135
100
E-Cad
135
100
100
N-Cad
135
100
Vimentin
65
Snail1
35
35
Slug
20
25
RhoA
20
45
β-actin
35
Fig. 2B (PANC-1-IN)

## Slide 4
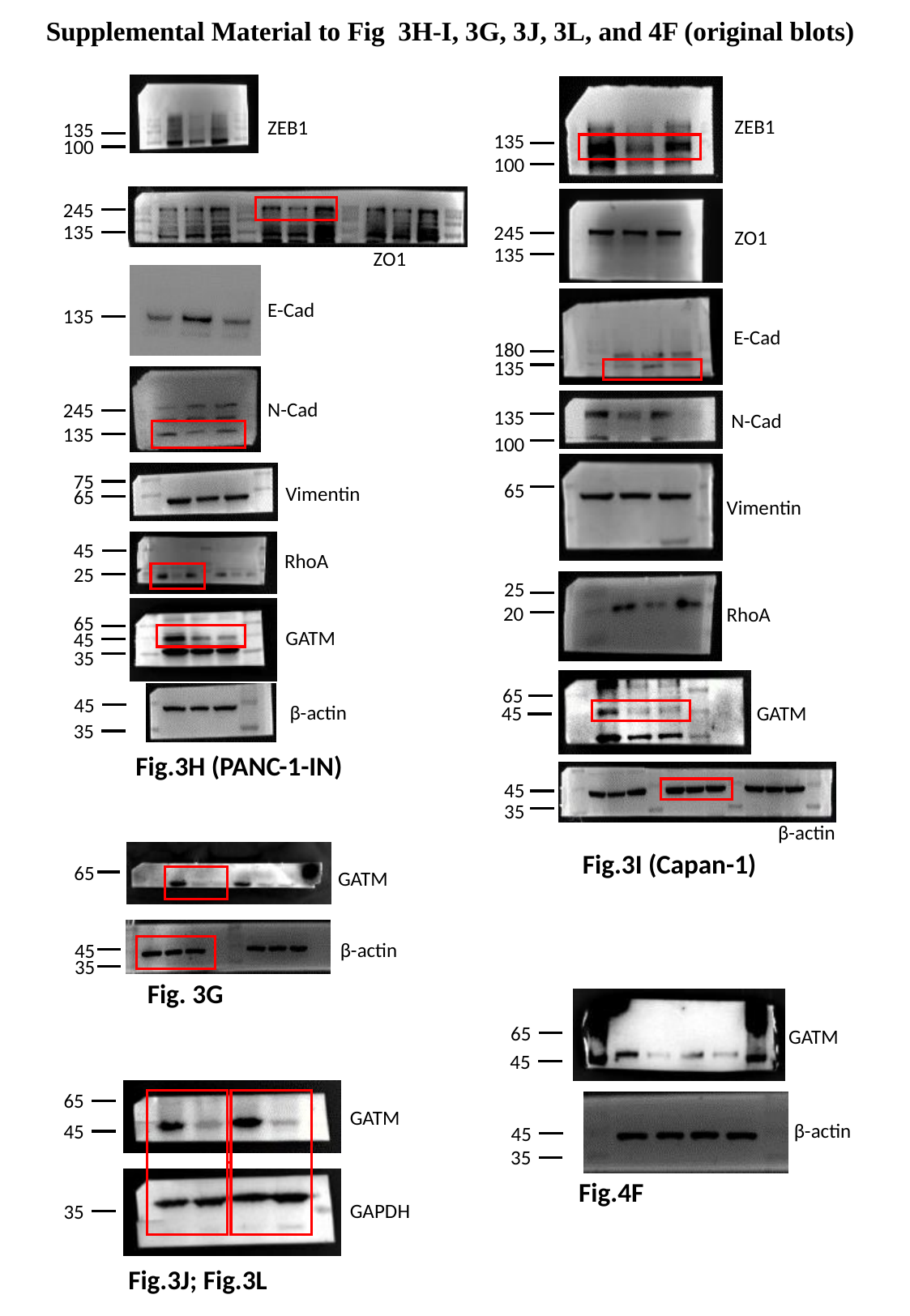

Supplemental Material to Fig 3H-I, 3G, 3J, 3L, and 4F (original blots)
ZEB1
135
100
245
135
ZO1
E-Cad
135
N-Cad
245
135
75
Vimentin
65
45
RhoA
25
65
GATM
45
35
β-actin
35
Fig.3H (PANC-1-IN)
45
ZEB1
135
100
245
ZO1
135
E-Cad
180
135
135
N-Cad
100
65
Vimentin
25
RhoA
65
GATM
45
35
β-actin
Fig.3I (Capan-1)
20
45
65
GATM
β-actin
35
Fig. 3G
45
65
GATM
45
β-actin
35
Fig.4F
45
65
GATM
45
GAPDH
35
Fig.3J; Fig.3L

## Slide 5
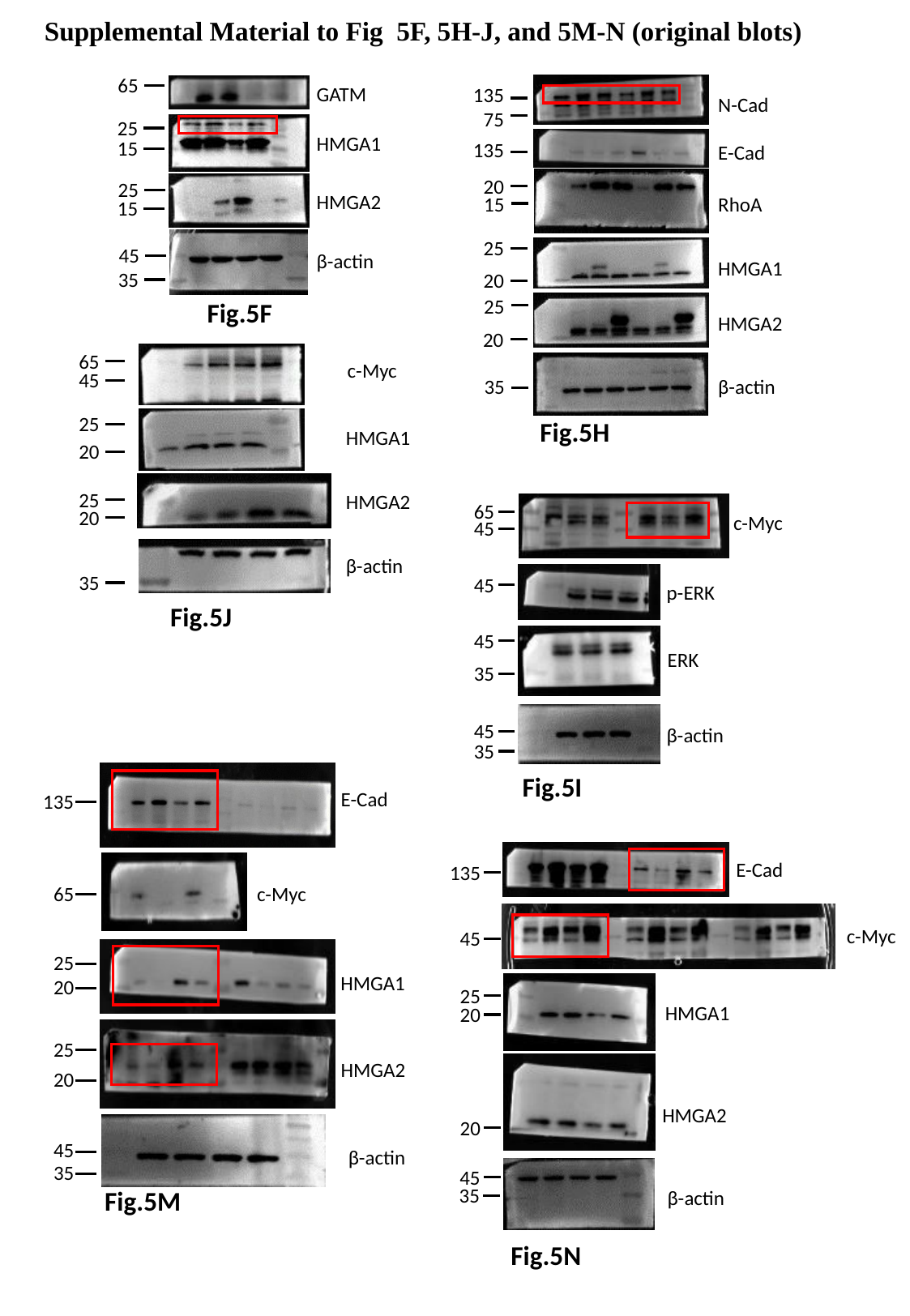

Supplemental Material to Fig 5F, 5H-J, and 5M-N (original blots)
65
GATM
25
HMGA1
15
25
HMGA2
15
β-actin
35
Fig.5F
45
135
N-Cad
75
135
E-Cad
20
RhoA
25
HMGA1
20
25
HMGA2
20
35
β-actin
Fig.5H
15
65
c-Myc
25
HMGA1
20
25
HMGA2
20
β-actin
35
Fig.5J
45
65
c-Myc
45
45
p-ERK
45
ERK
35
β-actin
35
Fig.5I
45
E-Cad
135
65
c-Myc
25
HMGA1
20
25
HMGA2
20
β-actin
35
Fig.5M
45
E-Cad
135
c-Myc
45
25
HMGA1
20
HMGA2
20
35
β-actin
Fig.5N
45

## Slide 6
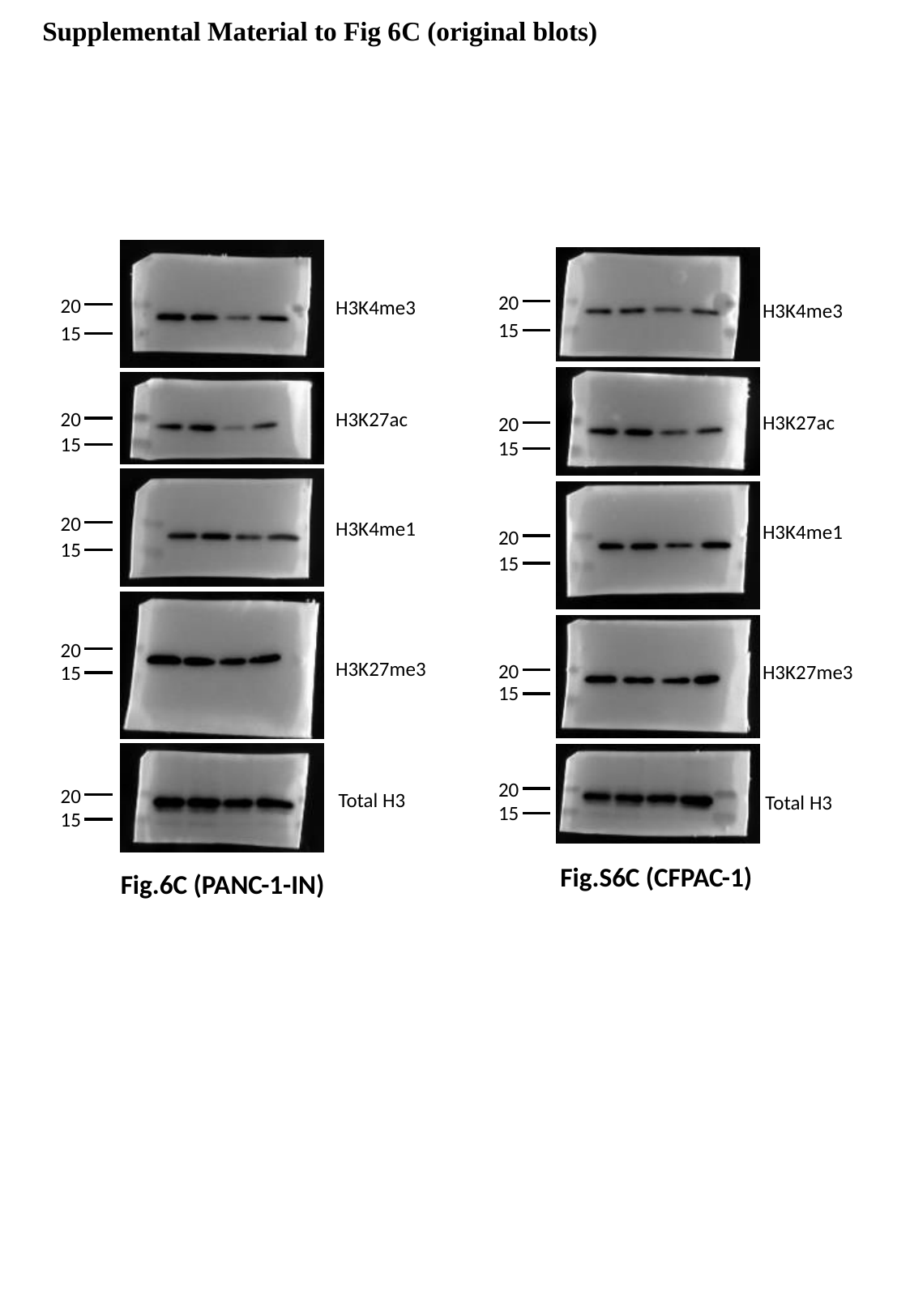

Supplemental Material to Fig 6C (original blots)
20
H3K4me3
15
H3K27ac
20
15
20
H3K4me1
15
20
H3K27me3
15
20
Total H3
15
Fig.6C (PANC-1-IN)
20
H3K4me3
15
H3K27ac
20
15
H3K4me1
20
15
20
H3K27me3
15
20
Total H3
15
Fig.S6C (CFPAC-1)

## Slide 7
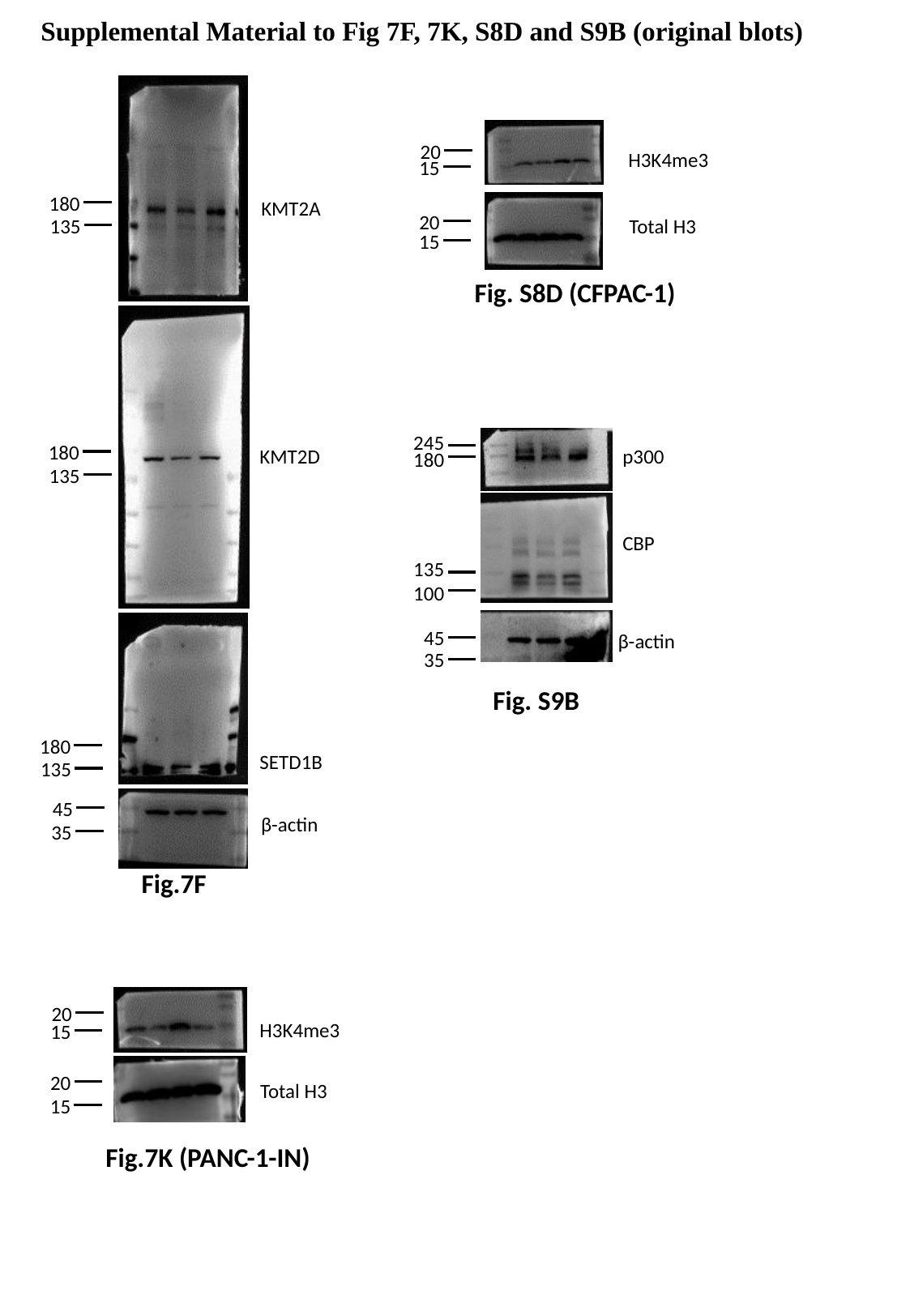

Supplemental Material to Fig 7F, 7K, S8D and S9B (original blots)
180
KMT2A
135
180
KMT2D
135
180
SETD1B
135
β-actin
35
Fig.7F
45
20
H3K4me3
15
20
Total H3
15
Fig. S8D (CFPAC-1)
245
p300
180
CBP
135
100
β-actin
35
Fig. S9B
45
20
H3K4me3
15
20
Total H3
15
Fig.7K (PANC-1-IN)

## Slide 8
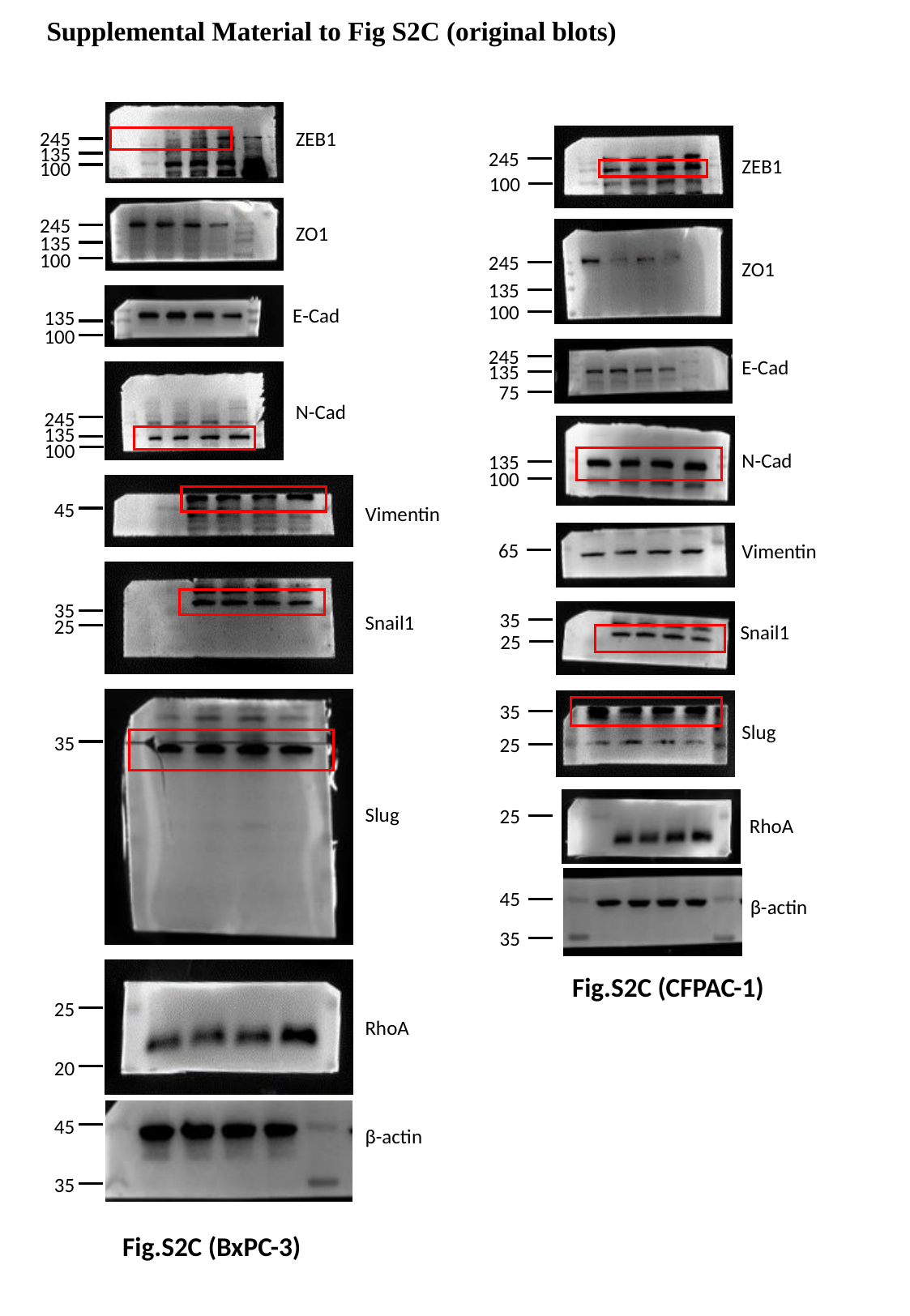

Supplemental Material to Fig S2C (original blots)
ZEB1
245
135
100
245
ZO1
135
100
E-Cad
135
100
N-Cad
245
135
100
45
Vimentin
35
Snail1
25
35
Slug
25
RhoA
20
45
β-actin
35
Fig.S2C (BxPC-3)
245
ZEB1
100
245
ZO1
135
100
245
E-Cad
135
75
N-Cad
135
100
65
Vimentin
35
Snail1
25
35
Slug
25
25
RhoA
β-actin
35
Fig.S2C (CFPAC-1)
45

## Slide 9
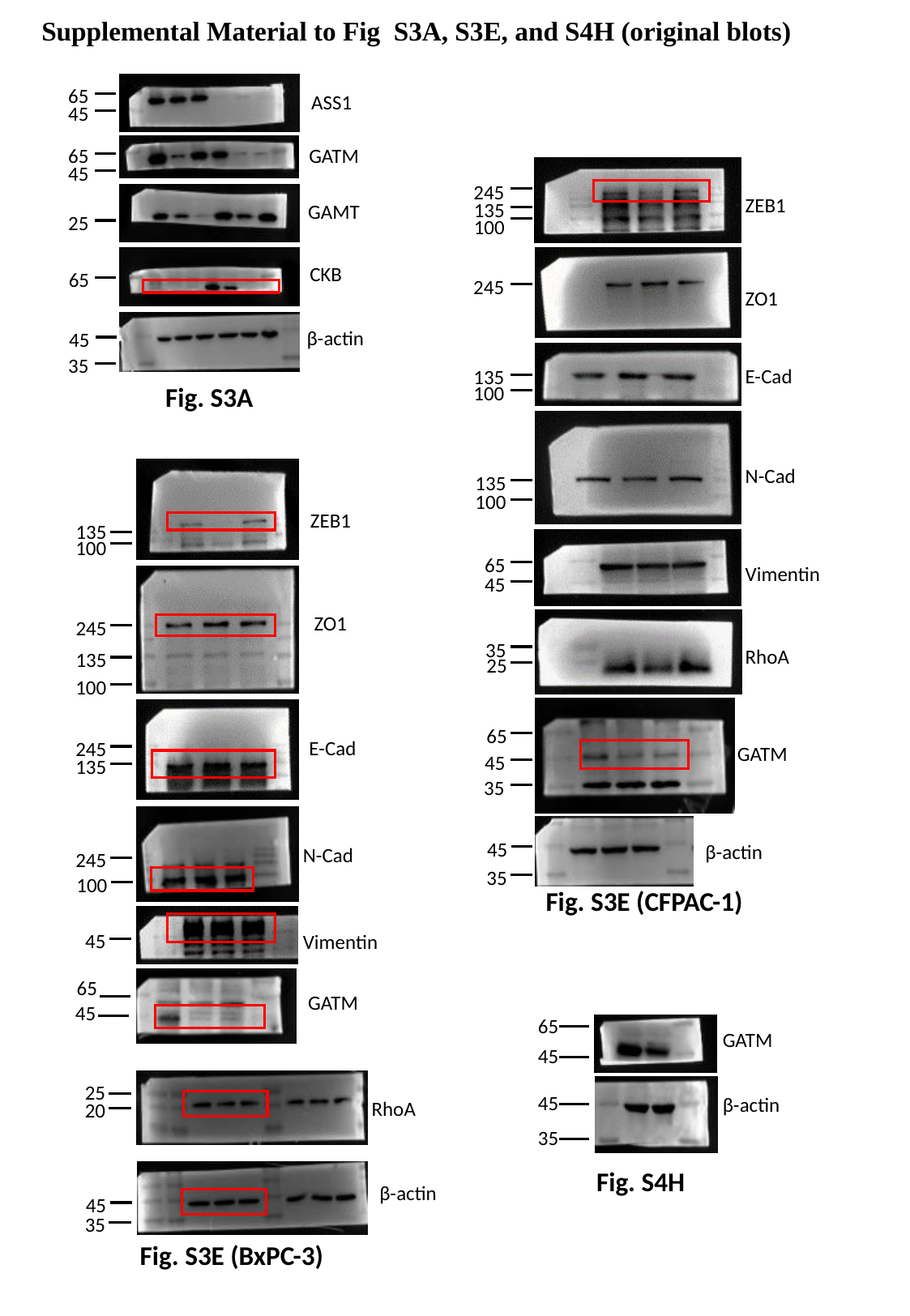

Supplemental Material to Fig S3A, S3E, and S4H (original blots)
65
ASS1
45
GATM
65
45
GAMT
25
CKB
65
β-actin
35
Fig. S3A
45
245
ZEB1
135
100
245
ZO1
E-Cad
135
100
N-Cad
135
100
65
Vimentin
45
35
RhoA
25
65
GATM
45
35
β-actin
35
Fig. S3E (CFPAC-1)
45
ZEB1
135
100
ZO1
245
135
100
E-Cad
245
135
N-Cad
245
100
45
Vimentin
65
45
GATM
25
RhoA
β-actin
35
Fig. S3E (BxPC-3)
20
45
65
GATM
45
β-actin
35
Fig. S4H
45

## Slide 10
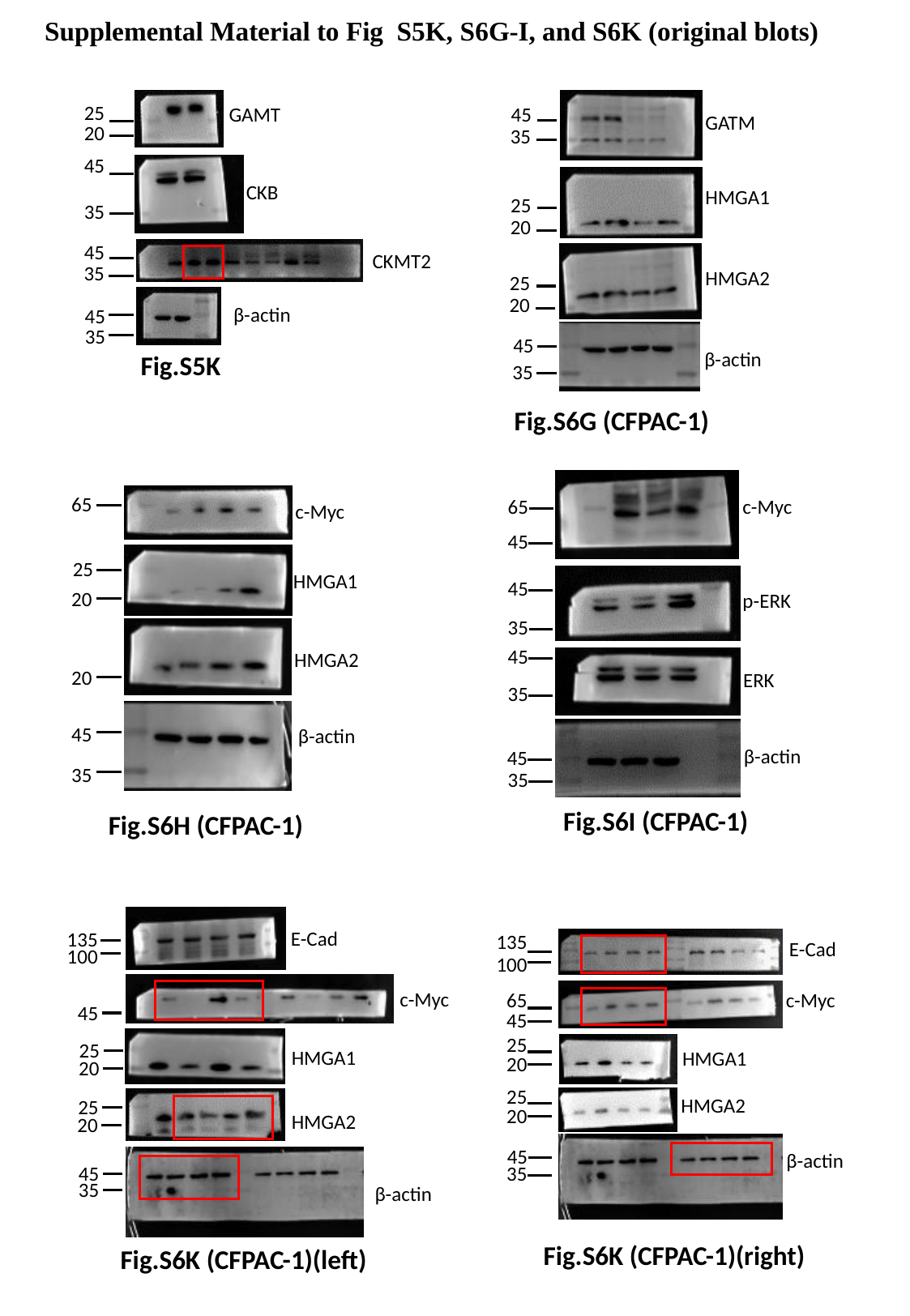

Supplemental Material to Fig S5K, S6G-I, and S6K (original blots)
45
GATM
35
HMGA1
25
20
HMGA2
25
20
β-actin
35
Fig.S6G (CFPAC-1)
45
25
GAMT
20
45
CKB
35
45
CKMT2
35
β-actin
35
Fig.S5K
45
65
c-Myc
45
45
p-ERK
35
45
ERK
35
β-actin
35
Fig.S6I (CFPAC-1)
45
65
c-Myc
25
HMGA1
20
HMGA2
20
45
β-actin
Fig.S6H (CFPAC-1)
35
E-Cad
135
100
c-Myc
45
25
HMGA1
20
25
HMGA2
20
35
β-actin
Fig.S6K (CFPAC-1)(left)
45
135
E-Cad
100
65
c-Myc
45
25
HMGA1
20
25
HMGA2
20
β-actin
35
Fig.S6K (CFPAC-1)(right)
45
