# Supplementary figures and images for: Epigenetic reprogramming-induced guanidinoacetic acid synthesis promotes pancreatic cancer metastasis and transcription-activating histone modifications
Source: J Exp Clin Cancer Res. 2023 Jun 28;42:155. doi: 10.1186/s13046-023-02698-x (PMC10304235; doi:10.1186/s13046-023-02698-x)

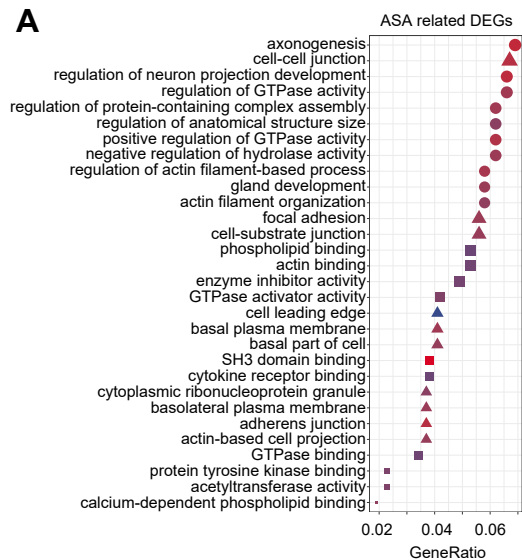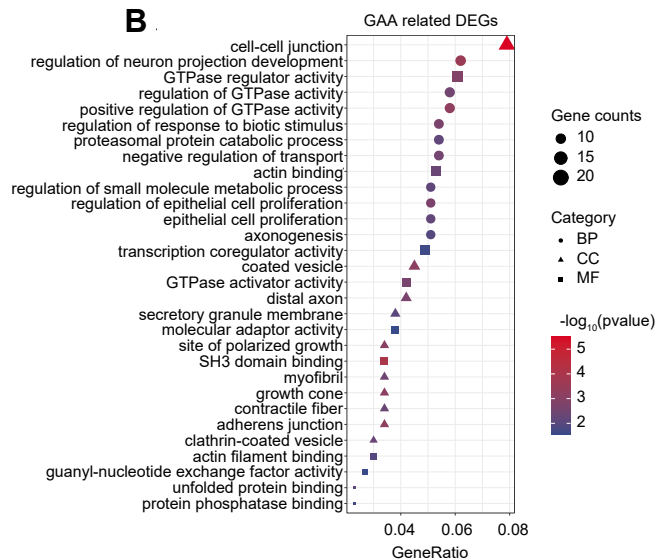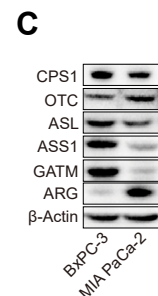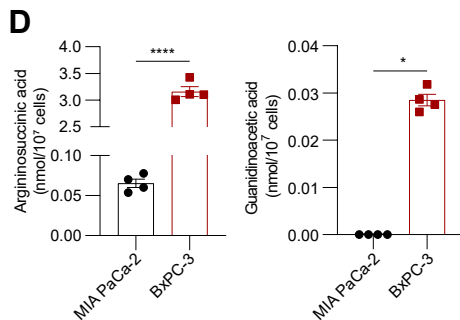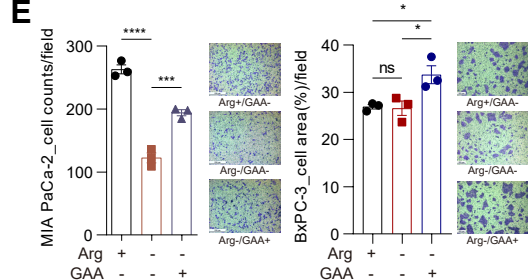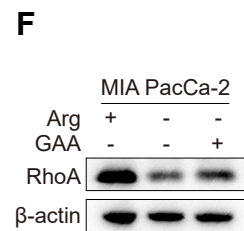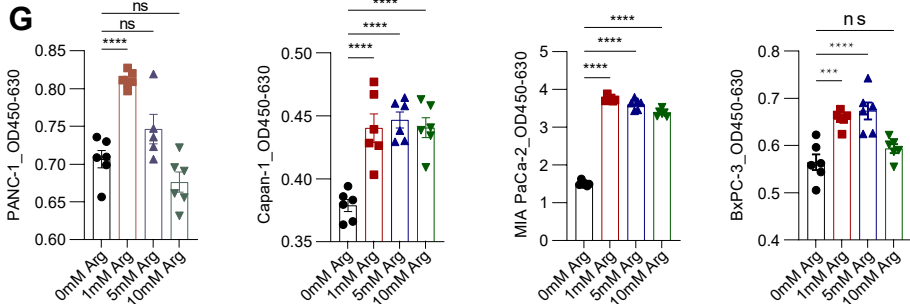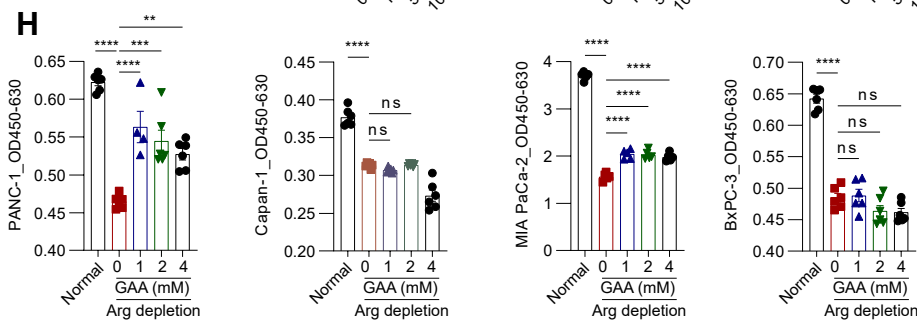

Supplement: Supplementary file 9 — Additional file 9: Fig. S1. A high level of intracellular GAA wasassociated with PDAC liver metastasis. Fig. S2. GAA promoted liver metastasis of PDAC. Fig. S3. Knockdown of GATM suppressed livermetastasis of PDAC. Fig. S4. Three-dimensional epigenomereprogramming upregulates GATM expression promoting PDAC metastasis. Fig. S5. Metabolic alteration after GAAmetabolism disturbance. Fig. S6. GAA metabolism promotes HMGA-induced EMTvia upregulated MYC expression. Fig. S7. GAA promotes H3K27ac modifications atcell cycle and apoptosis-related genes. Fig. S8. GAA promotes H3K4me3 ofmetastasis-related genes by upregulating histone methyltransferases expression. Fig. S9. GAA metabolism promotes H3K27acmodification at the enhancers of the MYC gene. [file 13046_2023_2698_MOESM9_ESM.zip › FigS1 Related to Fig. 1.pdf]

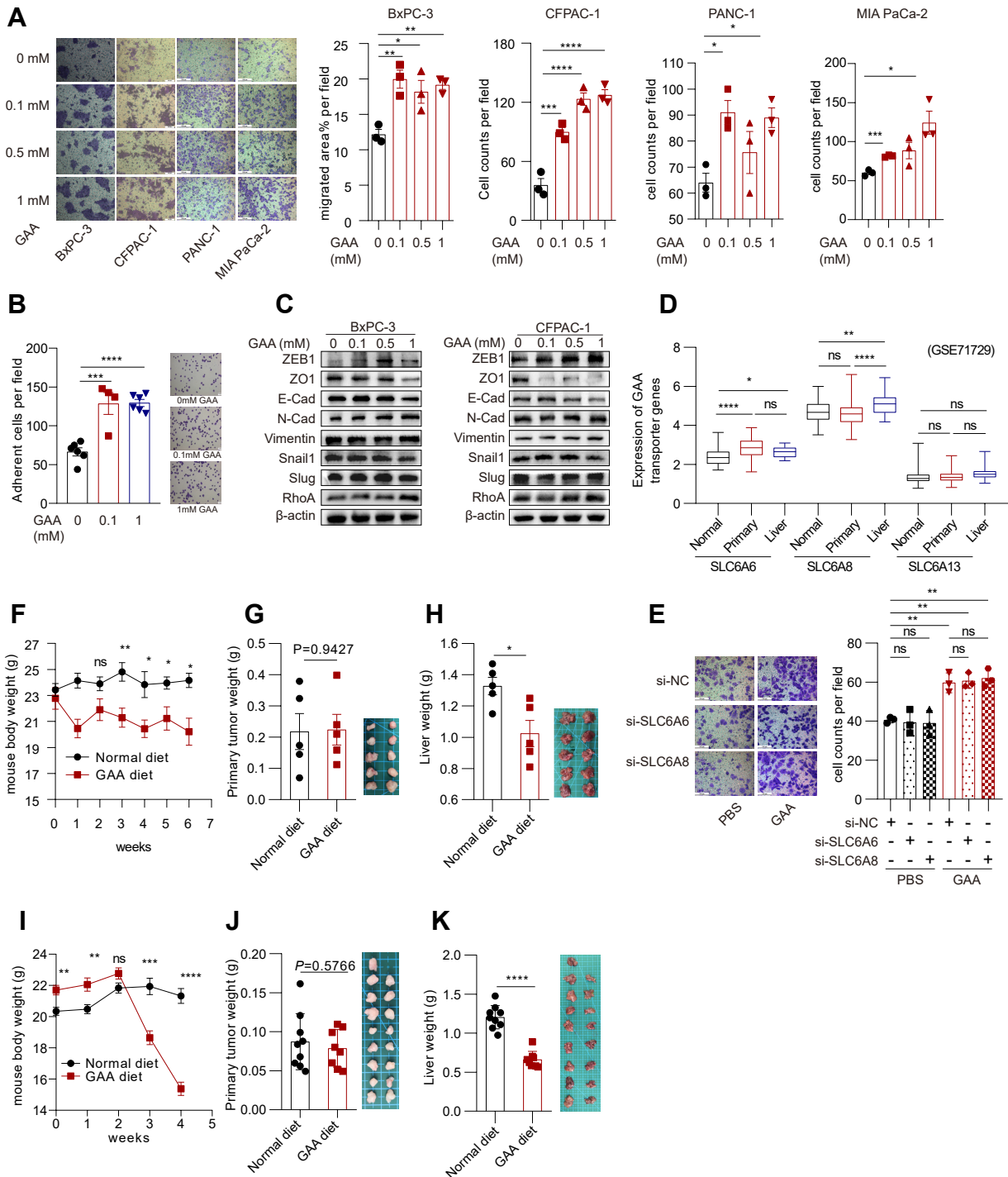

Supplement: Supplementary file 9 — Additional file 9: Fig. S1. A high level of intracellular GAA wasassociated with PDAC liver metastasis. Fig. S2. GAA promoted liver metastasis of PDAC. Fig. S3. Knockdown of GATM suppressed livermetastasis of PDAC. Fig. S4. Three-dimensional epigenomereprogramming upregulates GATM expression promoting PDAC metastasis. Fig. S5. Metabolic alteration after GAAmetabolism disturbance. Fig. S6. GAA metabolism promotes HMGA-induced EMTvia upregulated MYC expression. Fig. S7. GAA promotes H3K27ac modifications atcell cycle and apoptosis-related genes. Fig. S8. GAA promotes H3K4me3 ofmetastasis-related genes by upregulating histone methyltransferases expression. Fig. S9. GAA metabolism promotes H3K27acmodification at the enhancers of the MYC gene. [file 13046_2023_2698_MOESM9_ESM.zip › FigS2 Related to Fig. 2.pdf]

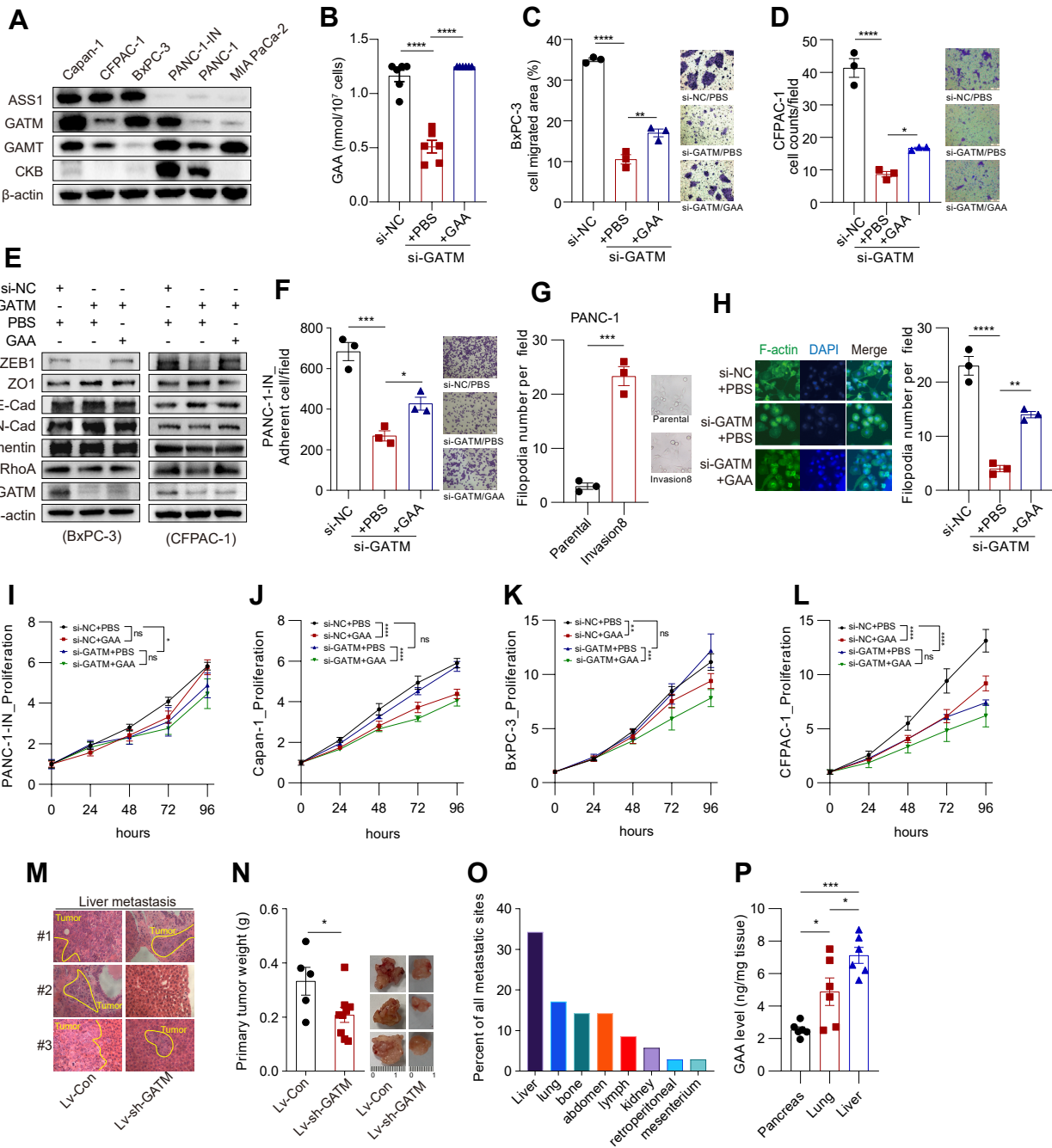

Supplement: Supplementary file 9 — Additional file 9: Fig. S1. A high level of intracellular GAA wasassociated with PDAC liver metastasis. Fig. S2. GAA promoted liver metastasis of PDAC. Fig. S3. Knockdown of GATM suppressed livermetastasis of PDAC. Fig. S4. Three-dimensional epigenomereprogramming upregulates GATM expression promoting PDAC metastasis. Fig. S5. Metabolic alteration after GAAmetabolism disturbance. Fig. S6. GAA metabolism promotes HMGA-induced EMTvia upregulated MYC expression. Fig. S7. GAA promotes H3K27ac modifications atcell cycle and apoptosis-related genes. Fig. S8. GAA promotes H3K4me3 ofmetastasis-related genes by upregulating histone methyltransferases expression. Fig. S9. GAA metabolism promotes H3K27acmodification at the enhancers of the MYC gene. [file 13046_2023_2698_MOESM9_ESM.zip › FigS3 Related to Fig. 3.pdf]

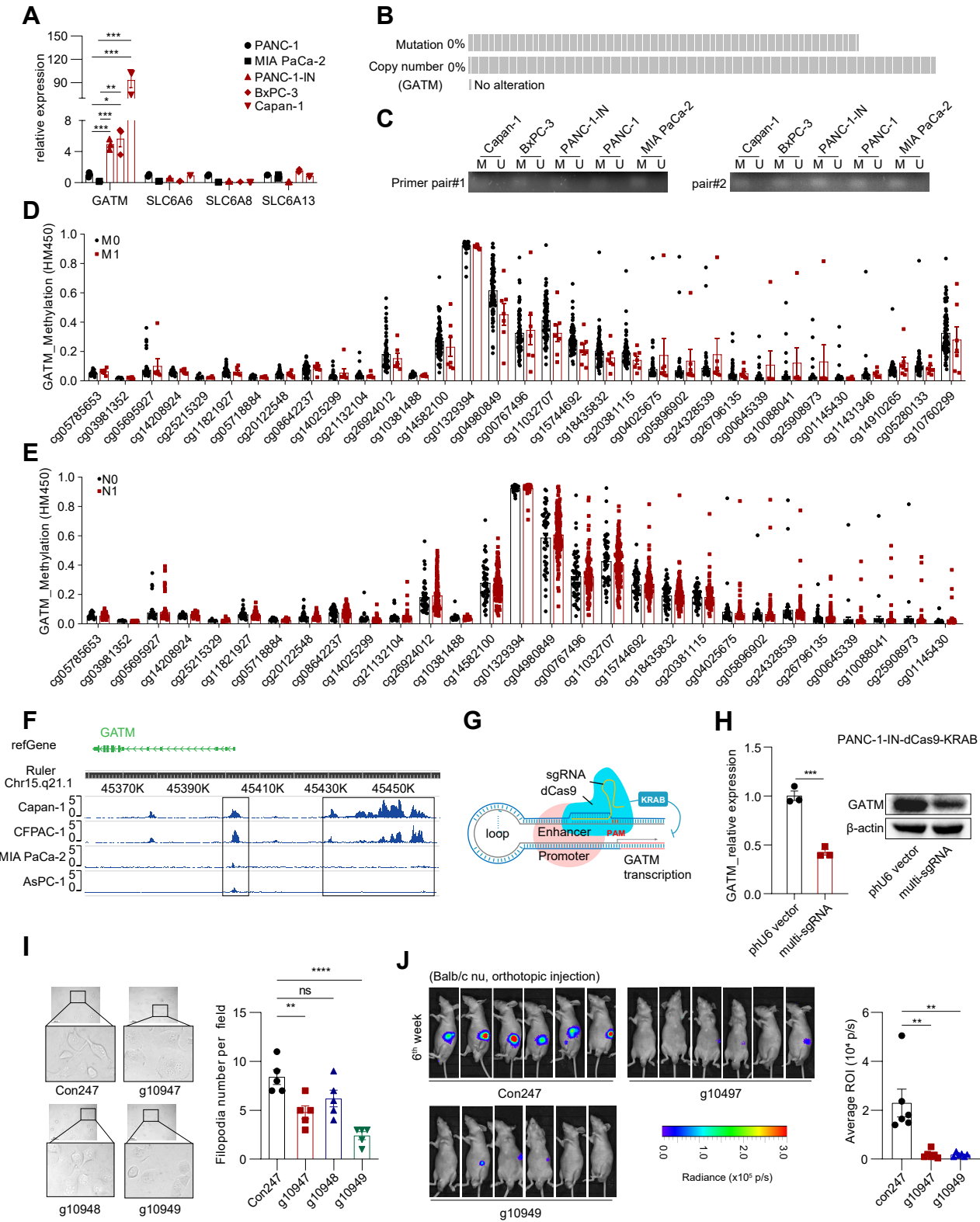

Supplement: Supplementary file 9 — Additional file 9: Fig. S1. A high level of intracellular GAA wasassociated with PDAC liver metastasis. Fig. S2. GAA promoted liver metastasis of PDAC. Fig. S3. Knockdown of GATM suppressed livermetastasis of PDAC. Fig. S4. Three-dimensional epigenomereprogramming upregulates GATM expression promoting PDAC metastasis. Fig. S5. Metabolic alteration after GAAmetabolism disturbance. Fig. S6. GAA metabolism promotes HMGA-induced EMTvia upregulated MYC expression. Fig. S7. GAA promotes H3K27ac modifications atcell cycle and apoptosis-related genes. Fig. S8. GAA promotes H3K4me3 ofmetastasis-related genes by upregulating histone methyltransferases expression. Fig. S9. GAA metabolism promotes H3K27acmodification at the enhancers of the MYC gene. [file 13046_2023_2698_MOESM9_ESM.zip › FigS4 Related to Fig. 4.pdf]

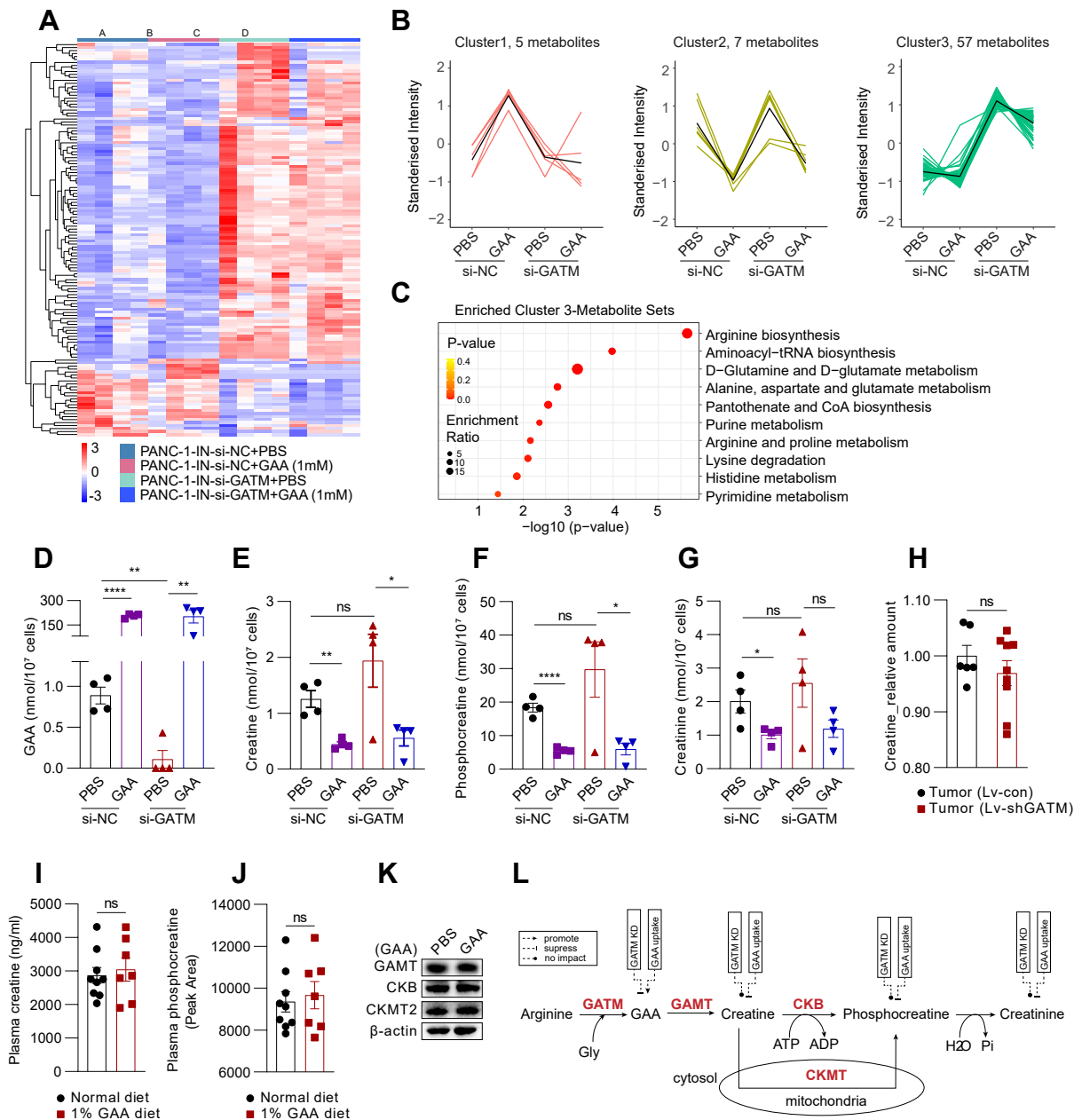

Supplement: Supplementary file 9 — Additional file 9: Fig. S1. A high level of intracellular GAA wasassociated with PDAC liver metastasis. Fig. S2. GAA promoted liver metastasis of PDAC. Fig. S3. Knockdown of GATM suppressed livermetastasis of PDAC. Fig. S4. Three-dimensional epigenomereprogramming upregulates GATM expression promoting PDAC metastasis. Fig. S5. Metabolic alteration after GAAmetabolism disturbance. Fig. S6. GAA metabolism promotes HMGA-induced EMTvia upregulated MYC expression. Fig. S7. GAA promotes H3K27ac modifications atcell cycle and apoptosis-related genes. Fig. S8. GAA promotes H3K4me3 ofmetastasis-related genes by upregulating histone methyltransferases expression. Fig. S9. GAA metabolism promotes H3K27acmodification at the enhancers of the MYC gene. [file 13046_2023_2698_MOESM9_ESM.zip › FigS5 Related to Fig. 5.pdf]

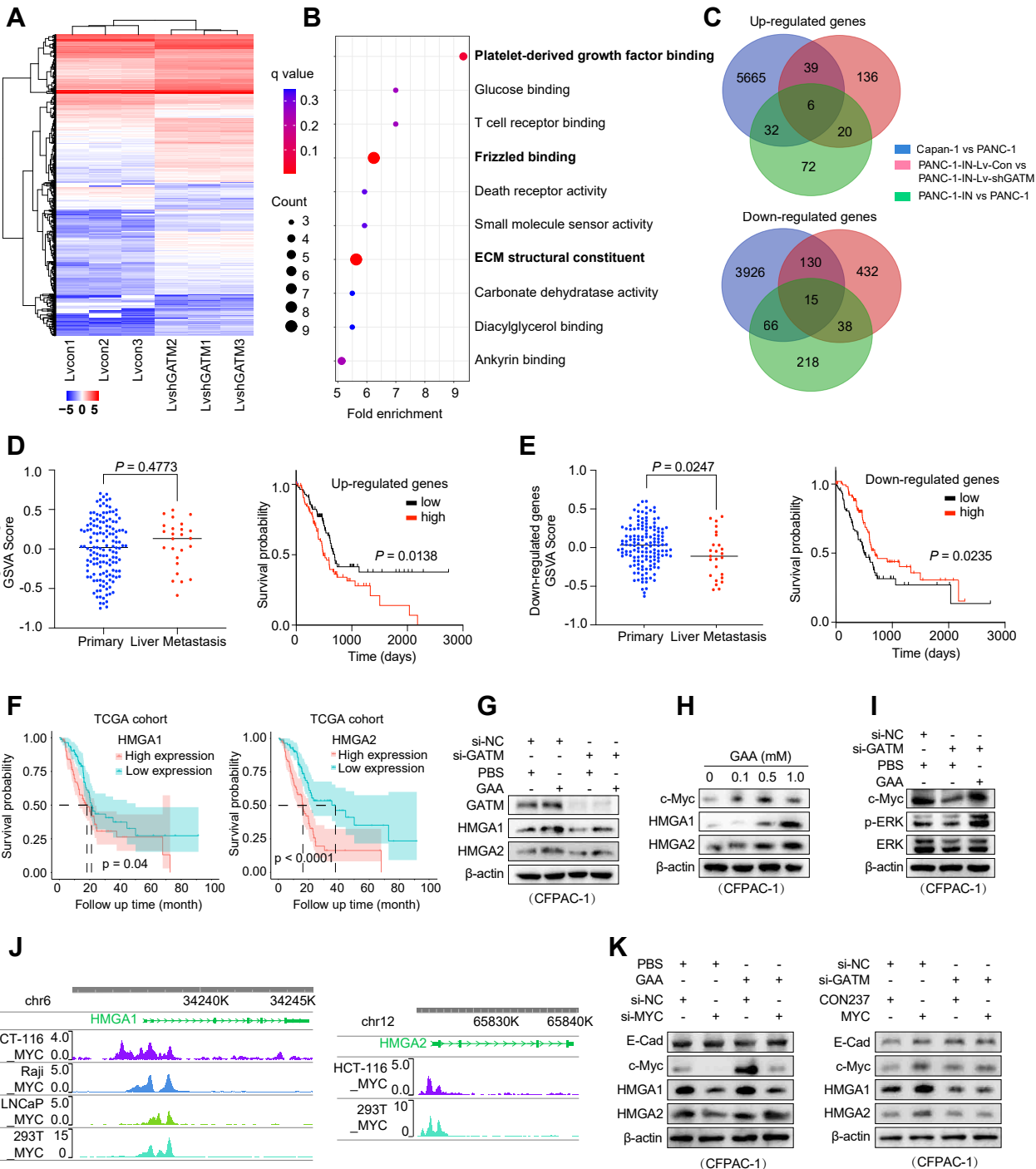

Supplement: Supplementary file 9 — Additional file 9: Fig. S1. A high level of intracellular GAA wasassociated with PDAC liver metastasis. Fig. S2. GAA promoted liver metastasis of PDAC. Fig. S3. Knockdown of GATM suppressed livermetastasis of PDAC. Fig. S4. Three-dimensional epigenomereprogramming upregulates GATM expression promoting PDAC metastasis. Fig. S5. Metabolic alteration after GAAmetabolism disturbance. Fig. S6. GAA metabolism promotes HMGA-induced EMTvia upregulated MYC expression. Fig. S7. GAA promotes H3K27ac modifications atcell cycle and apoptosis-related genes. Fig. S8. GAA promotes H3K4me3 ofmetastasis-related genes by upregulating histone methyltransferases expression. Fig. S9. GAA metabolism promotes H3K27acmodification at the enhancers of the MYC gene. [file 13046_2023_2698_MOESM9_ESM.zip › FigS6 Related to Fig. 5.pdf]

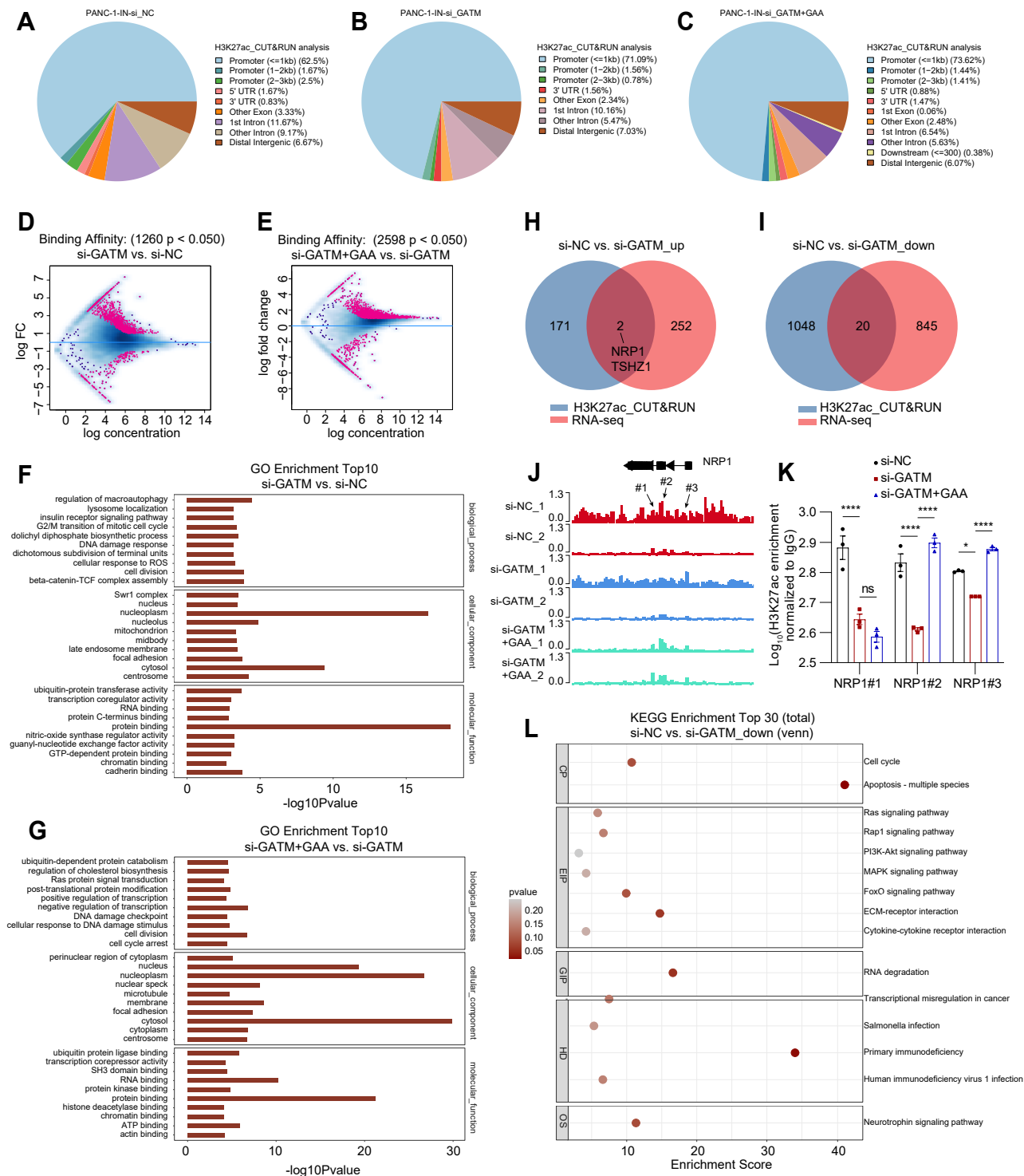

Supplement: Supplementary file 9 — Additional file 9: Fig. S1. A high level of intracellular GAA wasassociated with PDAC liver metastasis. Fig. S2. GAA promoted liver metastasis of PDAC. Fig. S3. Knockdown of GATM suppressed livermetastasis of PDAC. Fig. S4. Three-dimensional epigenomereprogramming upregulates GATM expression promoting PDAC metastasis. Fig. S5. Metabolic alteration after GAAmetabolism disturbance. Fig. S6. GAA metabolism promotes HMGA-induced EMTvia upregulated MYC expression. Fig. S7. GAA promotes H3K27ac modifications atcell cycle and apoptosis-related genes. Fig. S8. GAA promotes H3K4me3 ofmetastasis-related genes by upregulating histone methyltransferases expression. Fig. S9. GAA metabolism promotes H3K27acmodification at the enhancers of the MYC gene. [file 13046_2023_2698_MOESM9_ESM.zip › FigS7 Related to Fig. 6 .pdf]

**A**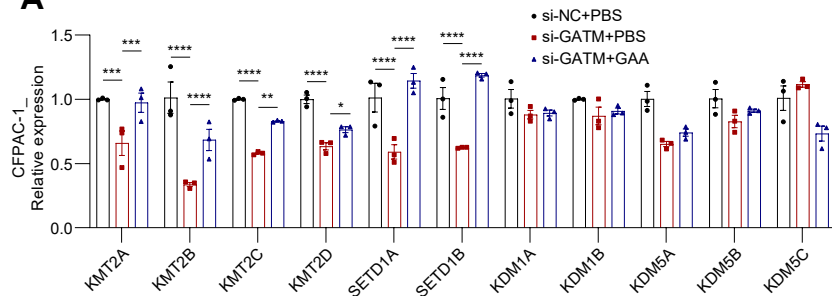**B**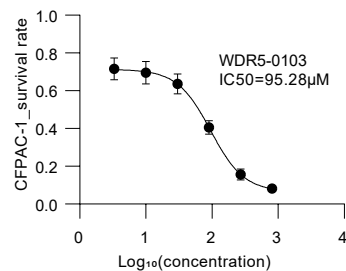**C**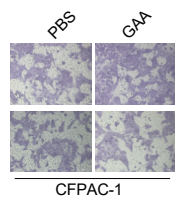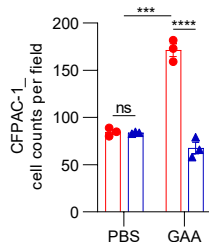**D**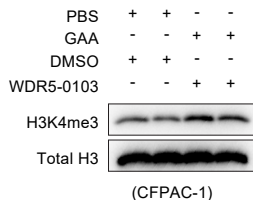**E**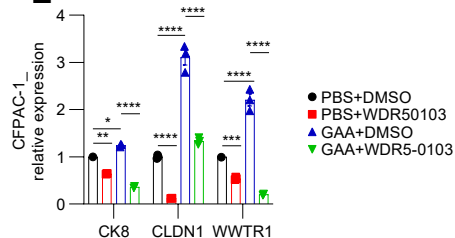**F**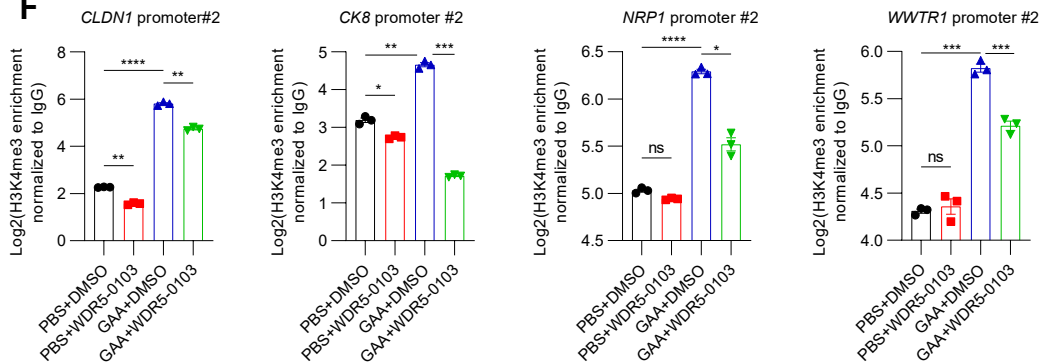

Supplement: Supplementary file 9 — Additional file 9: Fig. S1. A high level of intracellular GAA wasassociated with PDAC liver metastasis. Fig. S2. GAA promoted liver metastasis of PDAC. Fig. S3. Knockdown of GATM suppressed livermetastasis of PDAC. Fig. S4. Three-dimensional epigenomereprogramming upregulates GATM expression promoting PDAC metastasis. Fig. S5. Metabolic alteration after GAAmetabolism disturbance. Fig. S6. GAA metabolism promotes HMGA-induced EMTvia upregulated MYC expression. Fig. S7. GAA promotes H3K27ac modifications atcell cycle and apoptosis-related genes. Fig. S8. GAA promotes H3K4me3 ofmetastasis-related genes by upregulating histone methyltransferases expression. Fig. S9. GAA metabolism promotes H3K27acmodification at the enhancers of the MYC gene. [file 13046_2023_2698_MOESM9_ESM.zip › FigS8 Related to Fig. 7.pdf]

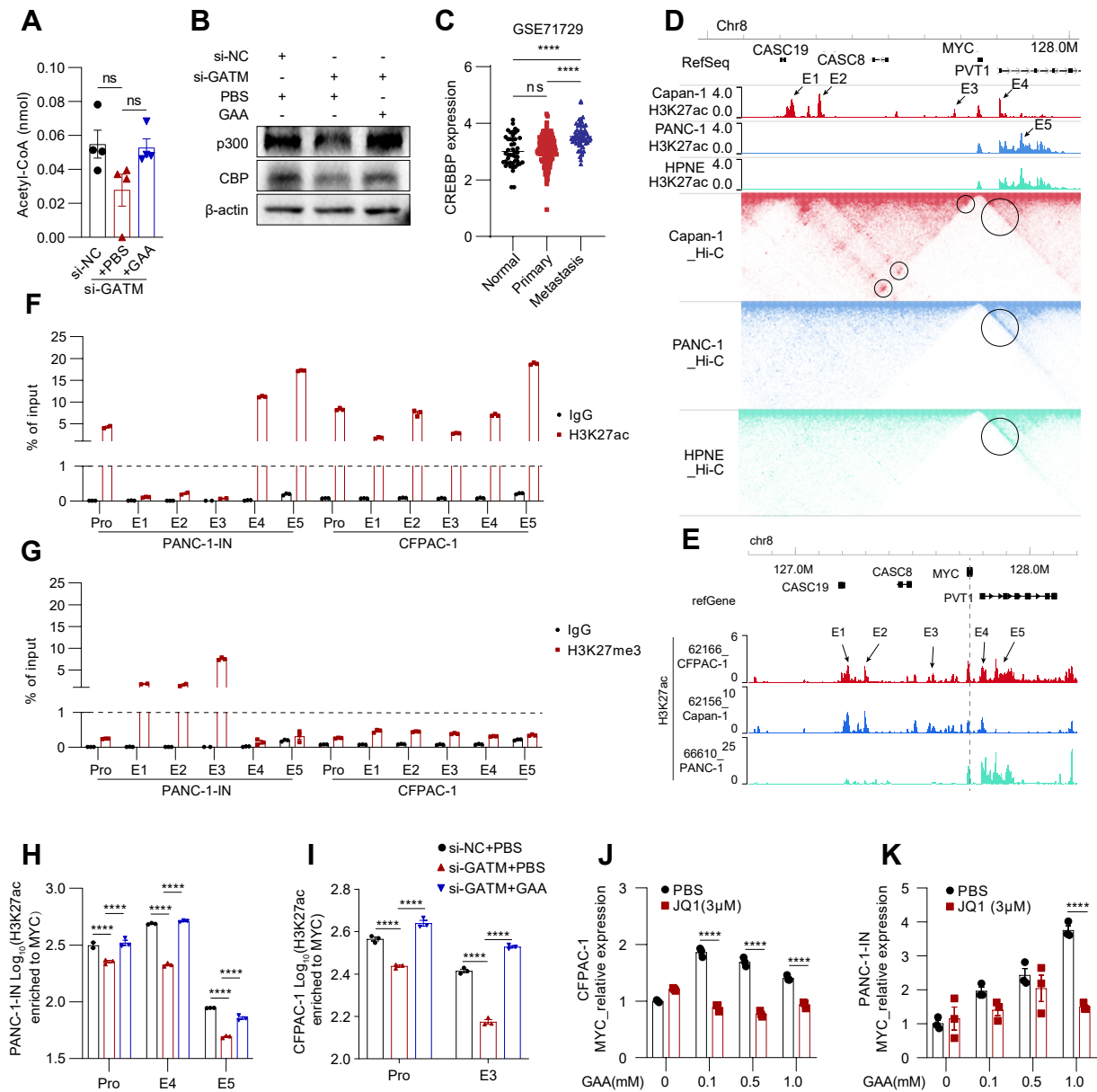

Supplement: Supplementary file 9 — Additional file 9: Fig. S1. A high level of intracellular GAA wasassociated with PDAC liver metastasis. Fig. S2. GAA promoted liver metastasis of PDAC. Fig. S3. Knockdown of GATM suppressed livermetastasis of PDAC. Fig. S4. Three-dimensional epigenomereprogramming upregulates GATM expression promoting PDAC metastasis. Fig. S5. Metabolic alteration after GAAmetabolism disturbance. Fig. S6. GAA metabolism promotes HMGA-induced EMTvia upregulated MYC expression. Fig. S7. GAA promotes H3K27ac modifications atcell cycle and apoptosis-related genes. Fig. S8. GAA promotes H3K4me3 ofmetastasis-related genes by upregulating histone methyltransferases expression. Fig. S9. GAA metabolism promotes H3K27acmodification at the enhancers of the MYC gene. [file 13046_2023_2698_MOESM9_ESM.zip › FigS9 Related to Fig. 7.pdf]
